# Supplementary material for: Deciphering the exact breakpoints of structural variations using long sequencing reads with DeBreak
Source: Nat Commun. 2023 Jan 17;14:283. doi: 10.1038/s41467-023-35996-1 (PMC9845341; doi:10.1038/s41467-023-35996-1)
Supplement: Supplementary file 2 — Editorial Assessment Report [file 41467_2023_35996_MOESM2_ESM.pdf]

nature portfolio GUIDED OPEN ACCESS

Contents of this report

1. **Manuscript details**: overview of your manuscript and the editorial team.
2. **Review synthesis**: summary of the reviewer reports provided by the editors.
3. **Editorial recommendation**: personalized evaluation and recommendation from all 3 journals.
4. **Annotated reviewer comments**: the referee reports with comments from the editors.
5. **Open research evaluation**: advice for adhering to best reproducibility practices.

About the editorial process

Because you selected the **Nature Portfolio Guided Open Access** option, your manuscript was assessed for suitability in three of our titles publishing high-quality work across the spectrum of methods research: ***Nature Methods***, ***Nature Communications***, and ***Communications Biology***. More information about Guided Open Access can be found here.

Collaborative editorial assessment

Your editorial team discussed the manuscript to determine its suitability for the Nature Portfolio Guided OA pilot. Our assessment of your manuscript takes into account several factors, including whether the work meets the technical standard of the Nature Portfolio and whether the findings are of immediate significance to the readership of at least one of the participating journals in the Guided OA pilot.

Peer review

Experts were asked to evaluate the following aspects of your manuscript:

- **Novelty** in comparison to prior publications;
- **Likely audience** of researchers in terms of broad fields of study and size;
- **Potential impact** of the study on the immediate or wider research field;
- **Evidence** for the claims and whether additional experiments or analyses could feasibly strengthen the evidence;
- **Methodological detail** and whether the manuscript is reproducible as written;
- Appropriateness of the **literature review**.

Editorial evaluation of reviews

Your editorial team discussed the potential suitability of your manuscript for each of the participating journals. They then discussed the revisions necessary in order for the work to be published, keeping each journal's specific editorial criteria in mind.

Journals in the Nature portfolio will support authors wishing to transfer their reviews and (where reviewers agree) the reviewers' identities to journals outside of Springer Nature.
If you have any questions about review portability, please contact our editorial office at guidedoa@nature.com.

nature portfolio GUIDED OPEN ACCESS

Manuscript details

Tracking number	Submission date	Decision date	Peer review type
GUIDEDOA-22-00392	**Jan 14, 2022**	**Feb 28, 2022**	Single-blind

**Manuscript title**<br>DeBreak: Deciphering the exact breakpoints of structural variations using long sequencing reads<br>**Preprint:** Available on ResearchSquare	**Author details**<br>Zechen Chong<br>**Affiliation:** University of Alabama at Birmingham

Editorial assessment team

**Primary editor**	**George Inglis**<br>Home journal: ***Communications Biology***<br>ORCID: **0000-0002-9069-5242**<br>Email: **george.inglis@us.nature.com**
**Other editors consulted**	**Lin Tang**<br>Home journal: ***Nature Methods***<br>ORCID: **0000-0002-6050-0424**<br><br>**Ilse Ariadna Valtierra Gutierrez**<br>Home journal: ***Nature Communications***<br>ORCID: **0000-0003-4128-5914**
**About your primary editor**	George received his PhD in Genetics and Molecular Biology from Emory University, where he studied mouse models of voltage-gated sodium channel dysfunction and epilepsy. He also has research experience in epigenomics and *in vitro* models of neuronal development. George joined the editorial team of *Communications Biology* in September 2020 and is based in the New York office.

Editorial assessment and review synthesis

**Editor's summary and assessment**	Here, the authors present DeBreak, a computational tool to identify and annotate structural variants (SVs) from long-read sequencing data. They benchmark this tool to three relevant long-read SV callers (Sniffles, pbsv, and cuteSV) using a mix of simulations and previously published datasets, representing input sequencing data generated from distinct methods (PacBio, Nanopore, etc). They report that DeBreak offers improved accuracy and resolution in identifying SVs and breakpoints, and integrate multiple case studies to show how this approach can identify distinct sets of SVs from existing tools.<br><br>While the editors jointly decided to send this manuscript out to review based on the consistent accuracy and resolution of DeBreak across multiple datasets, there were some concerns about the conceptual advance and variable performance of DeBreak in identifying novel SVs from distinct input datasets, which prohibited further consideration by *Nature Methods*.
**Editorial synthesis of reviewer reports**	While the reviewers find DeBreak to be of potential interest to the field, they raise several concerns regarding its accessibility to users, limited detail in the methods, and potential influence of factors such as sequencing data type or coverage on performance. Taken together, these points supported the initial concerns from *Nature Methods*.<br><br>However, *Nature Communications* would be interested in considering a revision that provides additional simulations for benchmarking DeBreak and that includes comparisons to Sniffles across all datasets (per Referees #3-4); we would also strongly encourage to include comparisons to PBHoney (per Referees #3-4). It would also be essential that DeBreak supports python3 (per Referees #1-2), and that you fully address all the technical and methodological concerns from all referees.<br><br>*Communications Biology* would also be interested in considering a revised manuscript that supports python3 (per Referees #1-2), elaborates on the Methods and data reporting (per all referees), evaluates how parameters like coverage and sequencing data type impact accuracy (Referees #2-4), and annotates repeat classes (Referee #1). It would also be important to incorporate the discussion points raised by each reviewer, and at a minimum discuss the limitations of the current simulation approach, as noted by Reviewers #3-4.

Editorial recommendation

Nature Methods<br><br>Revision not invited	Neither the conceptual advance nor advance in performance demonstrated is sufficient for publication in *Nature Methods.*
Nature Communications<br><br>Major revisions with extension of the work	*Nature Communication*s would be interested in considering a revision that provides additional simulations for benchmarking DeBreak and that includes comparisons to Sniffles across all datasets (per Referees #3-4); we would also strongly encourage to include comparisons to PBHoney (per Referees #3-4). It would also be essential that DeBreak supports python3 (per Referees #1-2), and that you fully address all the technical and methodological concerns from all referees.
Communications Biology<br><br>Major revisions	*Communications Biology* would be interested in considering a revision that supports python3 (per Referees #1-2), elaborates on the Methods and data reporting (per all referees), evaluates how parameters like coverage and sequencing data type impact accuracy (Referees #2-4), and annotate repeat classes (Referee #1). It would also be important to incorporate the discussion points raised by each reviewer, and at a minimum discuss the limitations of the current simulation approach, as noted by Reviewers #3-4.

Next steps

**Editorial recommendation 1:**	Our top recommendation is to revise and resubmit your manuscript to *Nature Communications.* We feel the additional experiments required are reasonable to address within a 6 month timeframe.
**Editorial recommendation 2:**	You may also choose to revise and resubmit your manuscript to *Communications Biology.* This option might be best if the requested experimental revisions are not possible/feasible at this time.
**Note**	As stated on the previous page *Nature Methods* is not inviting a revision at this time. Please keep in mind that the journal will not be able to consider any appeals of their decision through Guided Open Access.

Revision

To follow our recommendation, please upload the revised manuscript files using **the link provided in the decision letter**. Should you need assistance with our manuscript tracking system, please contact Adam Lipkin, our Nature Portfolio Guided OA support specialist, at guidedOA@nature.com.

Revision checklist

- ☐ Cover letter, stating to which journal you are submitting
- ☐ Revised manuscript
- ☐ Point-by-point response to reviews
- ☐ Updated Reporting Summary and Editorial Policy Checklist
- ☐ Supplementary materials (if applicable)

Submission elsewhere

If you choose not to follow our recommendations, you can still take the reviewer reports with you.

Option 1: Transfer to another Nature Portfolio journal
Springer Nature provides authors with the ability to transfer a manuscript within the Nature Portfolio, without the author having to upload the manuscript data again. To use this service, **please follow the transfer link provided in the decision letter.** If no link was provided, please contact guidedOA@nature.com.

> *Note that any decision to opt in to In Review at the original journal is not sent to the receiving journal on transfer. You can opt in to In Review at receiving journals that support this service by choosing to modify your manuscript on transfer.*

Option 2: Portable Peer Review option for submission to a journal outside of Nature Portfolio
If you choose to submit your revised manuscript to a journal at another publisher, we can share the reviews with another journal outside of the Nature Portfolio if requested. You will need to request that the receiving journal office contacts us at guidedOA@nature.com. We have included editorial guidance below in the reviewer reports and open research evaluation to aid in revising the manuscript for publication elsewhere.

Annotated reviewer reports

The editors have included some additional comments on specific points raised by the reviewers below, to clarify requirements for publication in the recommended journal(s). However, please note that all points should be addressed in a revision, even if an editor has not specifically commented on them.

Reviewer #1 information

**Expertise**	This reviewer has expertise in computational genomics and variant annotation methods.
**Editor's comments**	This reviewer finds DeBreak to be a powerful tool for identifying SVs, but highlights the need for better annotation of its performance by repeat classes and potential discrepancies with results from PAV.

Reviewer #1 comments

Section	Annotated Reviewer Comments
**Remarks to the Author: Overall significance**	Chen et al described DeBreak, a new structural variation (SV) caller for long sequence reads. DeBreak differs from the existing SV callers in its use of local reassembly, which I think is the right direction. The authors show that DeBreak outperforms other popular SV callers on both simulated and real datasets.
**Remarks to the Author: Impact**	In my opinion, this manuscript could be a fit to *Nature Communications*.<br>**While we appreciate the reviewer's input, all decisions regarding publication are solely made by editors.**
**Remarks to the Author: Strength of the claims**	**Major comments:**<br><br>1) Please make DeBreak support python3. Python2 has retired for more than a year. Users would question the long-term commitment to DeBreak if they see it support python2 only. Python2-only also makes it difficult for others to contribute to DeBreak as there will be fewer Python2 programmers in future.<br>**This point was also raised by Referee #2, and should be addressed for further consideration at *Nature Communications* and *Communications Biology*.**<br><br>2) It would be good to stratify the result by repeat classes. For example, what is the accuracy for ALUs, LINE1s, SVAs, STRs, VNTRs and non-repeats? I predict that the accuracy of every caller will be near perfect for ALUs and LINE1s and will drop a lot in VNTRs. It is rare to see such stratification in SV caller papers, but I think this is an important analysis and is likely to benefit this manuscript. The authors can run

	RepeatMasker/TRF on the longest allele to annotate repeats.<br>**This point should be addressed for further consideration at *Nature Communications* and *Communications Biology*.**<br><br>3) I am a little concerned with the low consistency between DeBreak and PAV. Could the authors compare PAV HG002 calls to GIAB? It would be important to understand the accuracy of PAV. Another option is to use dipcall for assembly-based SV calling. Dipcall is known to agree with GIAB well. Its accuracy is lower than read-based SV calls mostly due to different variant representations. Note that dipcall also generates confident regions like GIAB.<br>**This point should be addressed for further consideration at *Nature Communications* and *Communications Biology*.**<br><br>Minor comments:<br><br>4) What assembler is used for local assembly? Is it wtdbg2?<br>**Several referees also commented on some confusion about specific tools or versions of software. For the sake of reproducibility, please carefully expand on the Methods, and refer to the Open Research Evaluation at the bottom of this document for further guidance.**<br><br>5) Does DeBreak assemble all reads mapped to a candidate SV, or only assemble reads that contains the SV?<br><br>6) What is the tool and the command line for comparing SV callsets? Is it truvari?<br><br>7) The last two pages on both "Supplementary file"s are not properly formatted. I guess this is generated by PDF printing. It would be good to have Excel files instead as it is difficult to derive a text file from PDF.<br><br>*Signed review: Heng Li*
**Remarks to the Author: Reproducibility**	I could install and run DeBreak via Bioconda but I have not tried it on large-scale datasets.

Reviewer #2 information

**Expertise**	This reviewer has expertise in computational genomics and long-read sequencing.
**Editor's comments**	This reviewer also finds DeBreak to be a worthwhile method, but provides extensive feedback on making the code more accessible to potential users, and potential impacts of sequencing data type on performance. They also highlight the need to carefully proofread the manuscript for accuracy, in light of some potential overstatements.

Reviewer #2 comments

Section	Annotated Reviewer Comments
**Remarks to the Author: Overall significance**	Firstly my apologies to the authors for a slow review.<br><br>I have attempted to run DeBreak on some samples and have found it to be a useful tool. I hope that this tool will be used in future. However if the authors wish it to be used widely it will require some tuning. We were able to identify known events in samples using DeBreak that were not identified in either cuteSV or sniffles (VERSION 2). This is useful and I have already added DeBreak to my suite of tools to use to look for SVs.
**Remarks to the Author: Impact**	As I state below, I am already investigating using DeBreak to look at SVs alongside tools such as CuteSV and Sniffles. The analysis of SVs detected using long read technology is of huge interest and the tools available to do so are still maturing. DeBreak is a worthy tool in this suite of methods.<br>**While we appreciate the reviewer's input, we still feel the conceptual novelty and performance are not sufficient for further consideration by *Nature Methods*.**
**Remarks to the Author: Strength of the claims**	1) Installation 1:I do have several comments about the code and its implementation that have been challenging. Firstly, the code is written in python 2.7 - now end of life (not supported). Really the tool should be updated to a current version of python. Similarly, the dependencies required for installing are old with all having been updated in the last two years versus the tested versions described.<br>**This point was also raised by Referee #1, and should be addressed for further consideration at *Nature Communications* and *Communications Biology*.**

2) Installation 2: We also could not get the code to install using the suggested conda instructions - instead we had to install in a specific environment file (see below for the yaml file we created to make an environment). This may have been a peculiarity of our system but conda was unable to resolve the dependencies when creating an environment in the stepwise manner presented by the authors.

3) Incorrectly formatted VCF: The biggest concern was that we could not parse the VCF output using conventional tools such as bedtools intersect. The records as written are identified as being invalid.

> **This point should be addressed for further consideration at *Nature Communications*.**

Using the vcf_validator tool (from EBI) we see the following report for an example VCF file generated by DeBreak:

"According to the VCF specification, the input file is not valid
Error: INFO MAPQ does not match the meta specification Type=Integer (not in integer format). This occurs 795 time(s), first time in line 23.
Warning: A valid 'reference' entry is not listed in the meta section. This occurs 1 time(s), first time in line 23."

To understand this better, we did investigate the code. Overall the code is poorly documented and relies heavily on manual execution of tasks including compiling commands to run using os.system as well as manual writing of VCF files - the authors should consider using a library such as pysam to handle these functions to ensure compatibility. Essentially, the code could be significantly improved for both readability and speed. This is not essential for publication but it will be important for those seeking to use the tool in the future.

Ensuring the VCF file is the correct format is essential.

> **We strongly encourage you to better annotate the code for further consideration at *Nature Communications* and *Communications Biology*.**

4) Tool versioning: With respect to the manuscript itself, I found the text clear and easy to interpret. The authors should specify the benchmark software versions used for cuteSV, sniffles etc. This is particularly important as sniffles has recently been updated to version 2 and I assume the work presented here is with respect to an earlier version. Similarly cuteSV has been updated many times since its publication. For the record, neither cuteSV (1.0.13) or Sniffles (vs2) could detect the known breakpoint in our sample- which was detected by DeBreak.

> **Similar concerns were raised by other referees; please be sure to elaborate on the Methods section and clarify the rationale for each**

	**analytical step, as well as relevant versions of software.**<br><br>5) Overclaims: Some of the language in the manuscript requires moderation. The opening of the introduction argues that SVs play the major role in all human genomic variation. But my interpretation of the word "genomic" would exclude single nucleotide variation and so all one is left with is SVs. The following sentence argues that SVs contribute more diversity than any other type of variant - are the author talking at the population level? The individual level? Arguably we have a far better understanding of single nucleotide variants than we do SVs at the population scale. I agree with the overall point that the authors are making but they could moderate their claims without diluting the message or significance of the manuscript.<br>**This point was also raised by the other reviewers, please carefully qualify the manuscript to avoid any overstatements.**<br><br>6) Comparison with assembled genomes: The authors should also consider that the work of Chaisson et al on analysing SVs in CHM13 can now be directly compared with a completed genome.<br>**This point could simply be mentioned as a future direction for *Communications Biology*.**<br><br>7) Table Details: In Table S1 (genotyping accuracy), the authors show poorer performance for debreak in PacBIO HiFi reads than CLR. This is surprising - every other tool improves its performance as the read quality improves. The authors do not comment on this but it is very interesting - do they have an explanation for why this might be? Is DeBreak somehow optimised for noisier reads? This also contrasts with the F1 scores presented in table S2 - where HIFi does result in improved performance.<br>**This point should be addressed for further consideration at *Nature Communications* and *Communications Biology*.**
**Remarks to the Author: Reproducibility**	My comments in the field above address some reproducibility issues. In essence detailed comparisons require a correctly formatted VCF file.

Reviewers #3-4 information	
**Expertise**	These reviewers have expertise in computational biology, long-read sequencing, and genomics.
**Editor's comments**	These reviewers also find DeBreak to be a promising new tool, but note some weaknesses in the use of simulated datasets, and the need to evaluate how coverage or other parameters might influence results. These reviewers co-reviewed the manuscript, so you will note that their comments are identical.

Reviewers #3-4 comments	
**Section**	**Annotated Reviewer Comments**
**Remarks to the Author: Overall significance**	Chen et al. present DeBreak, a new method for the detection of structural variants (SVs) from third-generation long-read sequencing data. Overall, the paper is well-written and clear and the presented experiments demonstrate the performance of the new algorithm, which is benchmarked against three other algorithms (Sniffles, pbsv and cuteSV) using simulated long-read data and Nanopore and PacBio long-read data from a real human genomes, including HG002. Structural variant discovery is an important problem, and DeBreak is a valuable addition to the bioinformatics toolchain for SVs. The algorithm's ability to accurately determine the breakpoints of SVs at the sequence level has, besides generating biological insight, the advantage that it enables an integration of the SV calls into the reference panels used for downstream short-read-based SV genotypers (e.g. ParaGraph or potentially PanGenie).<br><br>Overall, we are positive about the publication of this paper. There are, however, important points that should be addressed prior to publication.<br><br>**Major:**<br><br>**- Abstract:**<br>a) The last sentence "DeBreak also demonstrates excellent performance in supplementing whole-genome assembly methods." should be toned down - it is unclear based on the presented results whether the DeBreak-unique calls (compared to assembly-based methods) are true- or false-positive calls.<br><br>- Simulations:<br>a) Simulations are always a good starting point, but the simulation approach chosen by the authors is simplistic. Structural variants tend to emerge around areas of existing sequence homology, i.e. not randomly, but in more repeat-rich

/ difficult-to-analyze regions of the human genome. An improved or second simulation experiment could be constructed e.g. by varying the copy number of existing repeats in the human genome. Alternatively, the simulations part could be left as it is, but its limitations would have to be made more clear in the section describing the results and in the Discussion.

It would be essential that you address this point with additional simulations and analysis for further consideration at *Nature Communications*. This point could be addressed via discussion of limitations for *Communications Biology*.

b) In the reduced coverage simulations part of the results, it would be important to assess the effect of coverage on SV breakpoint detection accuracy.

This point (and point c, below) would be necessary for further consideration at *Nature Communications* and *Communications Biology*.

c) DeBreak includes a specific component for the detection of long insertions. It would be important to better understand the specific contribution of this component to the algorithm's overall ability to detect insertions of different lengths (i.e., conditional on insertion length). Also, the paper includes a real-data evaluation of DeBreak's performance on insertions conditional on insertion length(see Figure 2); but even in the 50kb bin, DeBreak still achieves an F1 score of about 0.75. It would be important to better understand from which insertion size onwards the performance of DeBreak really starts breaking down; this could be assessed using simulations.

- HG002 results:

a) The authors highlight the importance of multi-allelic copy number variations (line 139) - what exactly is meant by "multi-allelic" here? Multi-allelic in one genome or in the human population? Also, if truth data are available multi-allelic copy number variants in HG002, it would be important to measure the accuracy of DeBreak's multi-allelic calls against these (genotype accuracy and breakpoint accuracy).

b) Downsampling: these results are very relevant; it would be important, however, to also understand the effect of reduced coverage on breakpoint detection accuracy.

This is similar to *Simulations: Point B* noted above.

c) Line 174: "Taken together, these results highlight that DeBreak can accurately identify different types of SVs with precise breakpoints in real human genomes.". This statement should be explicitly limited to insertions and deletions (which are the types of SV calls that were validated here).

As previously stated, please carefully qualify any claims to avoid overstatements.

- HGSVC results:
a) It would be important to also specify and discuss recall and precision of the different SV detection methods (Table S2 only specifies F1 scores). An additional table with these metrics could be added in which all evaluated samples (of one sequencing technology type) are combined.

b) Why was Sniffles not applied to these samples?

> **This point should be addressed for further consideration at *Nature Communications*.**

c) Why is no evaluation of SV genotype accuracy carried out on this dataset?

- Discussion:
a) It would be good to include a brief section on the effect of the sequencing read data type (CLR, HiFi, Nanopore) on accuracy.

b) While DeBreak can call insertions, deletions, inversions, and translocations, the empirical validation focuses on insertions and deletions (due to the availability of truth SV data). A brief paragraph discussing this and highlighting the fact that additional empirical validation of the algorithm's performance on inversions and translocations would be desirable should be added to the Discussion.

- Methods:
a) Overall the Methods section is too vague and not specific enough.

b) Raw SV signal detection: Please be more specific about how exactly "raw SV signals" are detected based on the alignments. Please provide details on the density-based clustering of SV signals.

c) It is unclear to us what happens to translocations during the SV detection and clustering phase; in particular, it is unclear to us how translocations between different chromosomes are detected / handled (as chromosomes are processed independently).

d) Large insertion detection via local assembly: How exactly is "enriched clipped alignment" defined? It is not clear to us how this step differs from the main raw SV signal detection step - which is also followed by a POA-based reassembly step? Also, it would seem to us that one key problem with long insertions is that the insertion may be longer than the read length - if we understand the approach of DeBreak correctly, however, the local re-assembly will only include reads that extend into the areas outside of the insertion (and thus miss the "middle region" of long insertions)? Last, but not least, how exactly is the local de novo assembly step carried out (e.g. using an external long-read assembly algorithm)?

Minor:

- General points:
a) PBHoney was mentioned in the introduction but is not evaluated - could the authors explicitly comment on why this is (or alternatively add it to the evaluations)?

> **We would strongly recommend that you address this point by including a comparison to PBHoney if possible for further consideration at *Nature Communications*. This point could be addressed textually, for *Communications Biology*.**

- Simulations:
a) There are 3 simulated datasets with varying read lengths, but Table 1 lumps them together - it would be good to see results broken down by read length (e.g. in the Supplement) to better understand potential impacts of read length. How exactly were translocations modeled (inter-chromosomal or across chromosomes)?

b) Adding a brief explanation where the different SV length simulation peaks in Figure S1 come from to the figure's legend would be helpful.

c) Figure S3b seems to show a systematic bias with respect to the breakpoint accuracy of DeBreak-determined duplications - could the authors comment on that?

d) Also, the red bars in Figure S3b are kind of hard to identify (apart from the outliers, see previous point) - consider modifying the graph to enable a better distinction between the variant types?

e) The simulations could be extended by a simulated Nanopore dataset; or a discussion point could be added explaining why the results of a Nanopore simulation would be expected to be similar to the PacBio simulation results.

f) Line 118, "81.33% of SVs with ±1bp shift around the SV breakpoint": This statistic include correctly determind (= 0bp shift) breakpoints, correct? Phrasing this as "within 1bp of the true SV breakpoint" may be more clear.

- HG002 results:
a) It is not exactly clear to us how genotype accuracy is calculated here. What are the possible "genotyes" in the truth set - just 0, 1 or 2, or does the truth also include copy-number-variant genotypes? Also, is genotyping accuracy calculated on the respective algorithm's call set, or on the complete call set (both ways to evaluate genotyping accuracy are important and should be included).

	b) Please also specify +-1bp breakpoint accuracy for all algorithms in the text (to make results fully comparable to the simulation experiment).<br><br>c) Figure 2: Please make the utilized read data type (CLR, HiFi, Nanopore) more explicit in the figure and the legend.<br><br>**- HGSVC results:**<br>a) Please also specify +-1bp breakpoint accuracy for all algorithms in the text (to make results fully comparable to the simulation experiment), and specfiy breakpoint accuracy as % of calls made<br><br>b) It would be interesting (though not essential) to see results for multi-allelic copy number variations here as well.<br><br>c) Line 207, "suggesting that alignment-based SV callers identified SV calls more consistently.": More consistently than assembly-based methods? As only one assembly-based data source (PAV) is considered here, this claim does not seem to follow.<br><br>**- Cancer genome results:**<br>a) A brief description of the input data (technology type, coverage, read lengths) in the main text would be very helpful.<br><br>b) Would it be possible to construct an assembly-based "truth set" here as well?<br><br>c) The fusion gene-specific validation approach leveraging IsoSeq data is very interesting. Could the same approach be applied to the results of the other SV calling methods (to the extent that they are capable of calling translocations) as well? (We would view this as very interesting, though not essential)<br><br>**- Methods:**<br>a) Explicitly mentioning the multi-allele step of the workflow may be helpful.<br><br>b) Line 312: "The density of SV raw signal is computed for each position on the chromosome (Supp fig)" -> Figure number missing.<br><br>c) Section 3.33 only mentions the PacBio CLR dataset, omitting the Nanopore and HiFi datasets (which are also downsampled).<br><br>d) Line 342: Please define when exactly a read is counted as "supporting"

Open research evaluation

Data citation
Please cite (within the main reference list) any datasets stored in external repositories that are mentioned within their manuscript. For previously published datasets, we ask that you cite both the related research article(s) and the datasets themselves. For more information on how to cite datasets in submitted manuscripts, please see our data availability statements and data citations policy: https://www.nature.com/documents/nr-data-availability-statements-data-citations.pdf<br><br>Citing and referencing data in publications supports reproducible research, by increasing the transparency and provenance tracking of data generated or analysed during research. Citing data formally in reference lists also helps facilitate the tracking of data reuse and may help assign credit for individuals' contributions to research. A number of Springer Nature imprints are signatories of the Joint Declaration on Data Citation Principles, which stress the importance of data resources in scientific communication.
**Code availability and citation**
Thank you for making your custom code available via Github. Upon publication, Nature Portfolio journals consider it best practice to release custom computer code in a way that allows readers to repeat the published results. Code should be deposited in a DOI-minting repository such as Zenodo, Gigantum or Code Ocean and cited in the reference list following the guidelines described in our policy pages (see link below). Authors are encouraged to manage subsequent code versions and to use a license approved by the open source initiative.<br><br>See here for more information about our code availability policies: https://www.nature.com/nature-portfolio/editorial-policies/reporting-standards#availability-of-computer-code
**Ethics**
We believe that authors, peer reviewers and editors should be required to disclose any competing interests that might influence their decisions and conclusions around a particular piece of content. In the interests of transparency and to help readers form their own judgements of potential bias, Nature Portfolio journals require authors to declare any competing financial and/or non-financial interests in relation to the work described.<br><br>Please provide a 'Competing interests' statement using one of the following standard

sentences:

1. The authors declare the following competing interests: [specify competing interests]
2. The authors declare no competing interests.

See the Nature Portfolio competing interests policy for further information:
https://www.nature.com/nature-research/editorial-policies/competing-interests
The Springer Nature policy can be found here:
https://www.springernature.com/gp/policies/editorial-policies

We believe that research that involves the use of clinical, biomedical or biometric data from human participants must only be carried out with the explicit consent of those whose data are involved. Consent must be obtained without any form of coercion and with participants' explicit understanding of the purpose for which their data will be used.

Please reiterate in the Methods section any ethics information from source datasets used in this study, involving human or animal participants.

Materials availability

Oligo sequences, concentrations of antibodies, and sources of cell lines must be included in the Methods (these can also be provided in a main Table and cited in the Methods). Please see the Nature Portfolio policy page for further details:
https://www.nature.com/commsbio/editorial-policies/reporting-standards#availability-of-materials
