# Supplementary material for: Deciphering the exact breakpoints of structural variations using long sequencing reads with DeBreak
Source: Nat Commun. 2023 Jan 17;14:283. doi: 10.1038/s41467-023-35996-1 (PMC9845341; doi:10.1038/s41467-023-35996-1)
Supplement: Supplementary file 3 — Supplementary File 1 [file 41467_2023_35996_MOESM3_ESM.pdf]

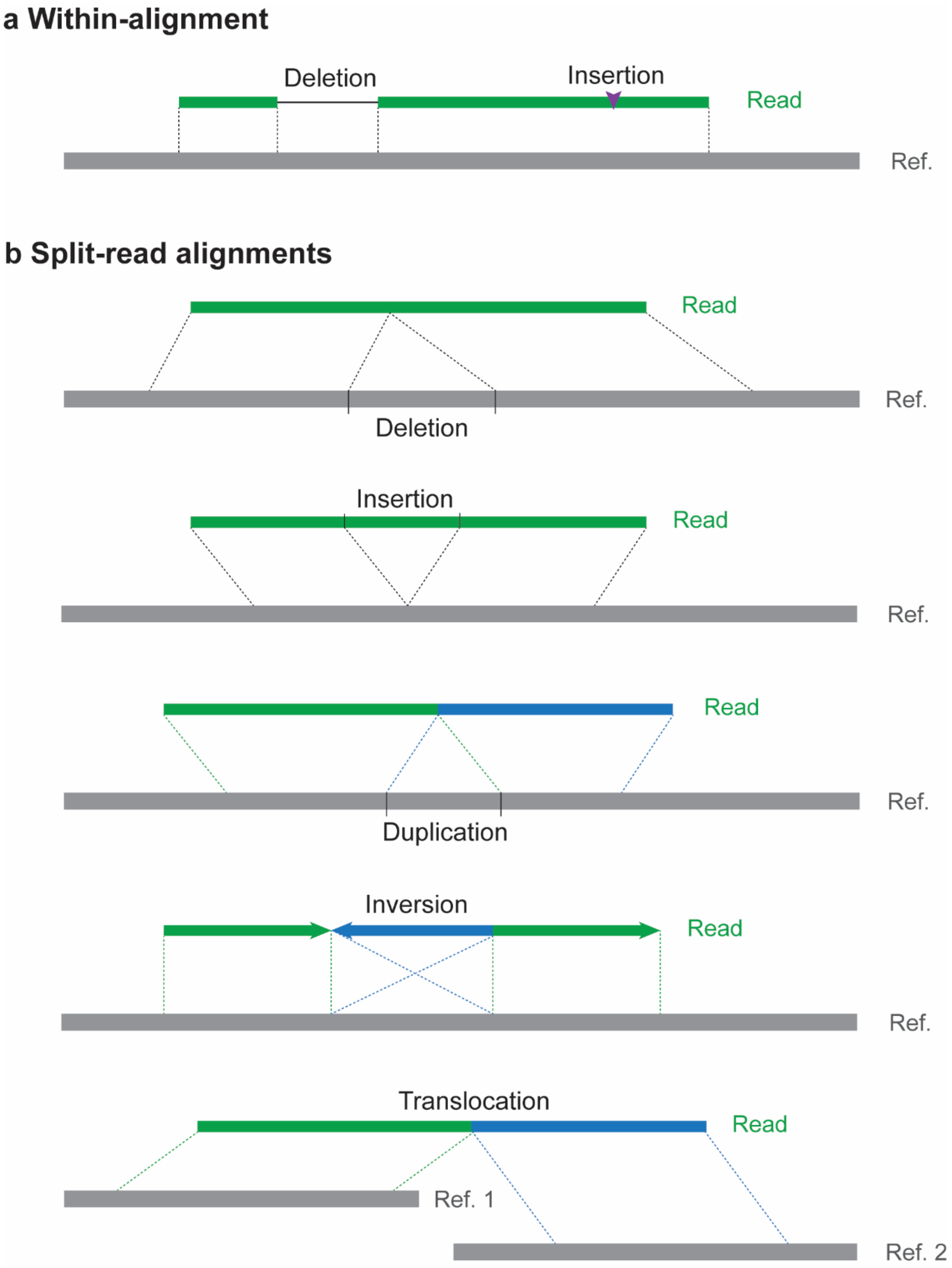

Figure S1 SV raw signal detection of DeBreak. a Deletion and insertion can be directly inferred within a single-read alignment. **b** Larger deletion and insertion, duplication, inversion, and translocation can be inferred from split-read alignments based on the location and orientation of two alignment segments. Alignments with distinct colors represent separate alignments of the same sequencing read.

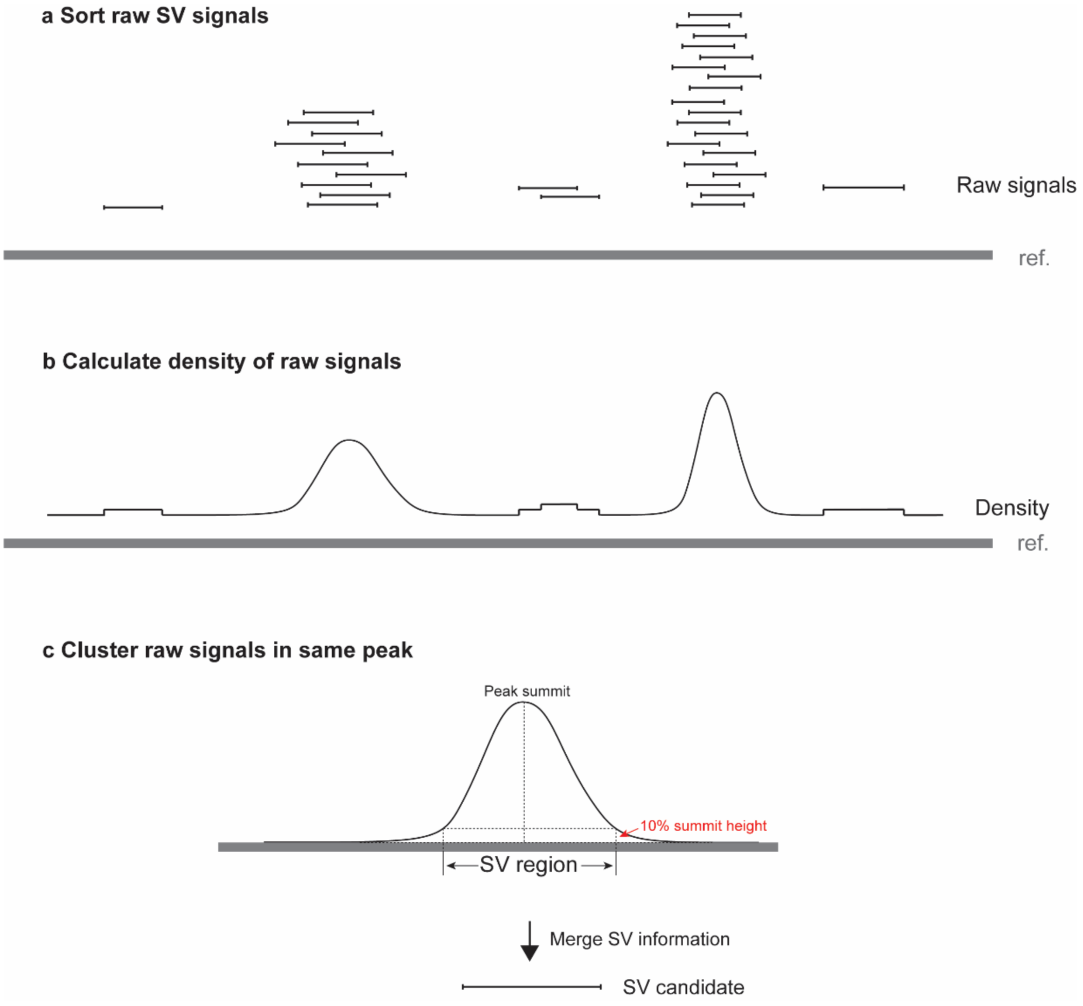

Figure S2 SV Density-based SV raw signal clustering. a SV raw signals from the same chromosome with the same SV type are sorted based on coordinates. **b** Density of raw signals is calculated for each base pair on the reference genome. DeBreak scans through the chromosome for peaks above a defined threshold. **c** For each peak, boundaries of the SV region for a SV event are determined where density drops to 10% of the peak summit height. All raw signals located within the SV region are merged into one SV candidate.

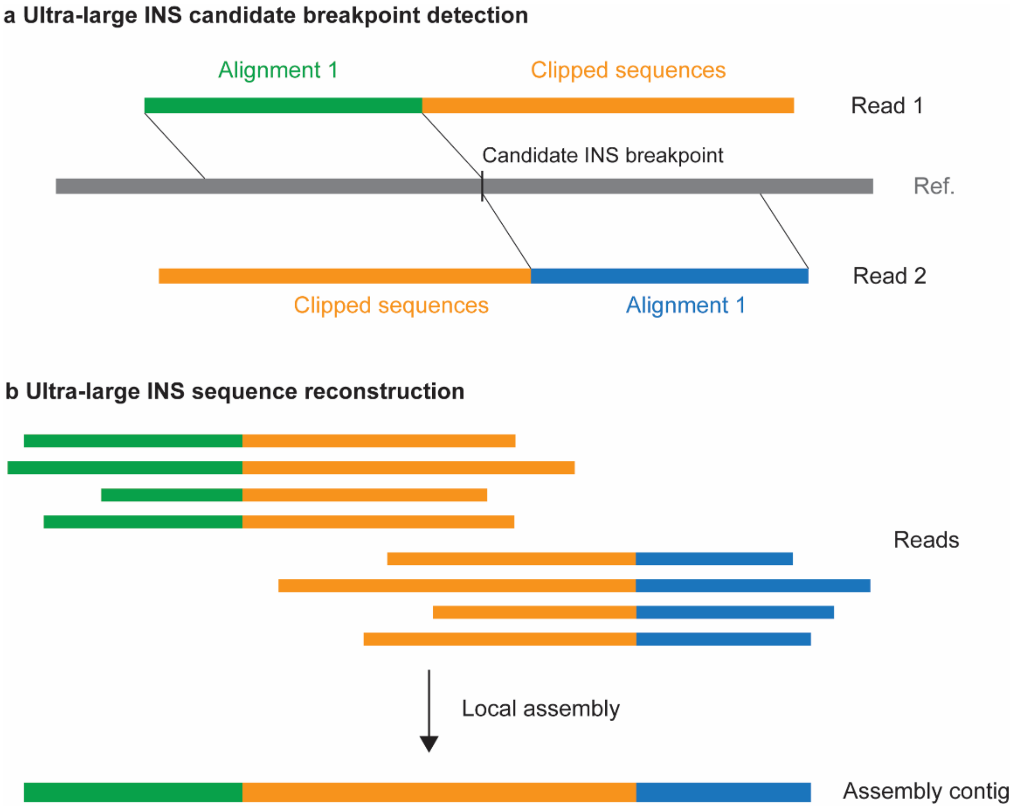

Figure S3 Ultra-large INS detection. a Example of reads with clipped alignment. Enriched “clipped” reads are required for both side of the candidate INS breakpoint for following local assembly. **b** Local *de novo* assembly using “clipped” reads. Reads aligned to both sides of the candidate INS breakpoint are collected for local assembly to generate an assembly contig that includes the full-length insertion sequence.

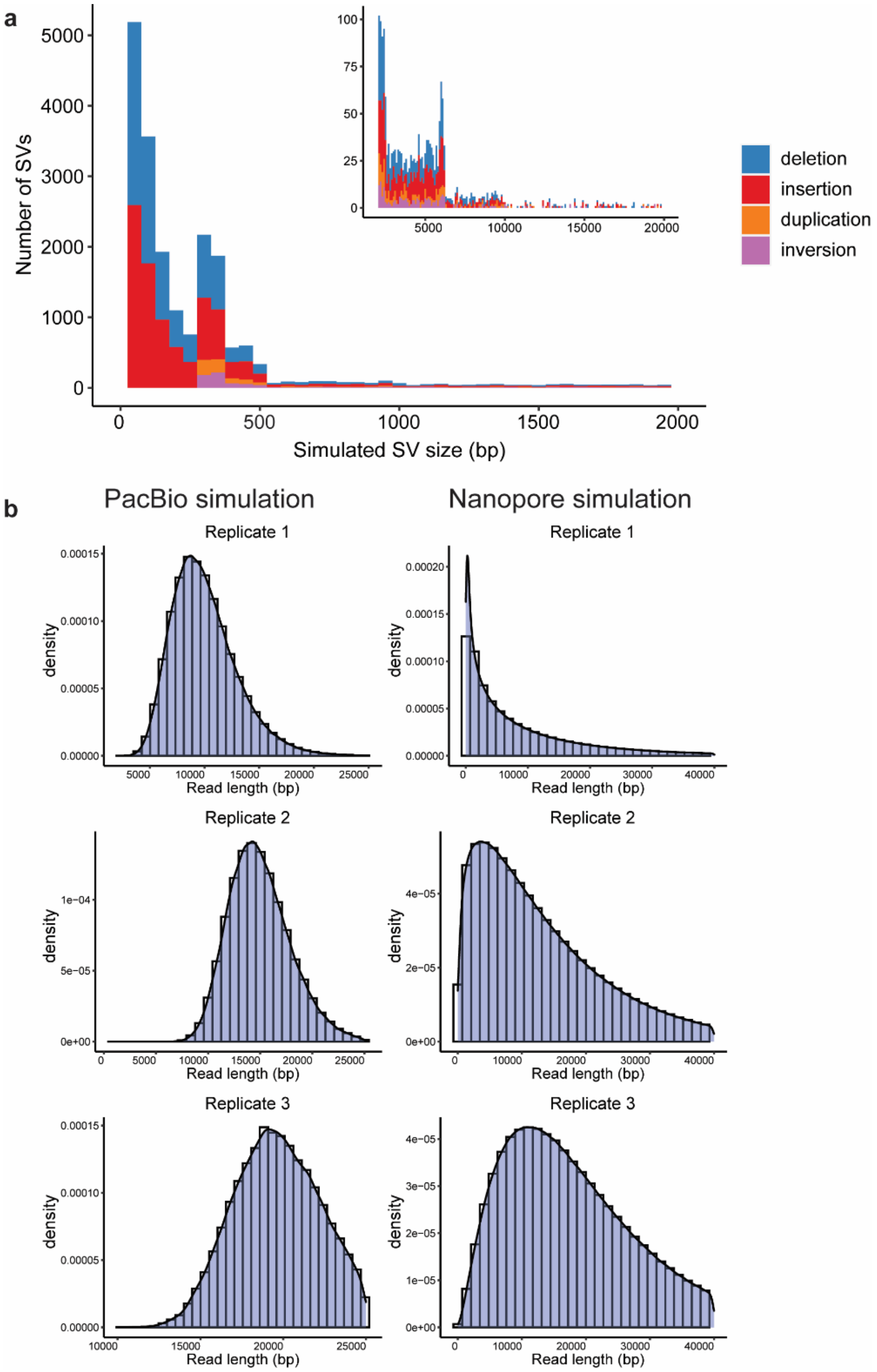

Figure S4 Characteristics of simulated datasets. a Size distribution of simulated SVs. Peaks at 300-350bp were simulated to mimic Alu elements, and peaks near 6kbp were simulated to mimic LINE mobile elements. **b** Length distributions of simulated PacBio (left) and Nanopore (right) reads in three simulated datasets.

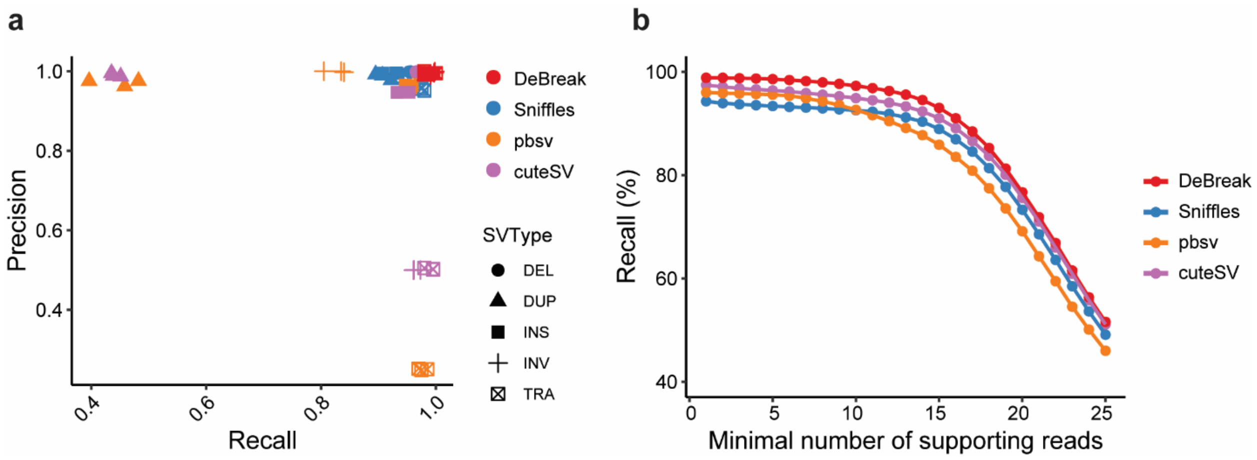

Figure S5 SV discovery accuracy in simulated PacBio datasets. a SV discovery accuracy for four tested SV callers in three simulated datasets. **b** Recall for deletion and insertion detection at different thresholds of 'minimal supporting reads' for the four SV callers in simulated datasets.

Table S1 SV discovery accuracy (F1 score) on three replicated simulated datasets

	DeBreak			Sniffles			pbsv			cuteSV		
**Type**	**Rep1**	**Rep2**	**Rep3**	**Rep1**	**Rep2**	**Rep3**	**Rep1**	**Rep2**	**Rep3**	**Rep1**	**Rep2**	**Rep3**
**PacBio**												
DEL	**99.54**	**99.53**	**99.56**	97.56	97.60	97.69	98.07	98.49	98.59	98.19	98.40	98.48
INS	**98.89**	**99.09**	**99.16**	94.95	96.25	96.20	95.58	96.13	95.69	93.96	94.96	95.01
DUP	**98.40**	**98.49**	**98.44**	94.11	94.90	94.63	64.52	62.06	56.33	60.54	61.91	60.71
INV	**99.65**	**99.05**	**99.85**	95.79	95.68	96.16	89.20	91.01	91.21	65.75	65.82	66.06
TRA	**99.50**	**99.75**	**98.99**	96.55	96.77	96.53	39.63	39.96	40.00	66.67	66.78	66.67
**Total**	**99.20**	**99.26**	**99.34**	96.15	96.78	96.81	94.12	94.58	94.32	92.53	93.12	93.16
**Nanopore**												
DEL	98.52	98.38	98.45	96.83	98.38	98.45	**98.74**	**98.59**	**98.47**	98.12	98.04	98.06
INS	**99.03**	**98.85**	**98.88**	96.02	98.85	98.88	96.63	96.45	96.36	94.97	95.13	94.94
DUP	**95.00**	**95.39**	**95.42**	94.89	95.39	95.42	62.07	57.37	61.25	55.72	57.43	57.00
INV	94.99	**94.57**	**95.55**	**95.01**	94.57	95.55	93.06	94.29	93.79	66.03	65.99	66.19
TRA	94.18	**98.48**	**95.83**	**95.15**	98.48	95.83	39.92	40.04	40.50	66.67	66.67	66.55
**Total**	**98.40**	**98.29**	**98.35**	96.28	98.29	98.35	94.97	94.77	94.78	92.89	92.97	92.89

The unit for the F1 score is %. The highest F1 score in each replicate is shown in bold.
Rep1, replicate 1 (10kbp). Rep2, replicate 2 (15kbp). Rep3, replicate 3 (20kbp).

Table S2 SV discovery accuracy of SV involving repeats

	Deletion			Insertion			Total		
Type	Recall	Precision	F1	Recall	Precision	F1	Recall	Precision	F1
**PacBio**									
DeBreak	**99.03**	99.86	**99.44**	**97.69**	98.12	**97.91**	**98.36**	98.99	**98.67**
Sniffles	97.72	99.86	98.78	95.19	97.85	96.49	96.46	98.85	97.64
Pbsv	98.13	99.97	99.04	88.95	**98.26**	93.37	93.54	**99.15**	96.26
cuteSV	97.97	**99.97**	98.96	94.77	97.06	95.89	96.37	98.52	97.43
**Nanopore**									
DeBreak	98.12	**98.51**	**98.31**	**97.35**	96.89	**97.12**	**97.73**	97.69	**97.71**
Sniffles	97.65	97.98	97.81	93.01	**98.21**	95.52	95.33	**98.09**	96.68
Pbsv	**99.13**	97.25	98.18	92.60	97.95	95.20	95.87	97.58	96.72
cuteSV	98.31	97.95	98.13	93.87	97.60	95.70	96.09	97.78	96.93

Averages for three replicates (10kbp, 15kbp,20kbp). The unit for recall, precision, and F1 score is %. The highest recall, precision, and F1 score for each category are shown in bold.

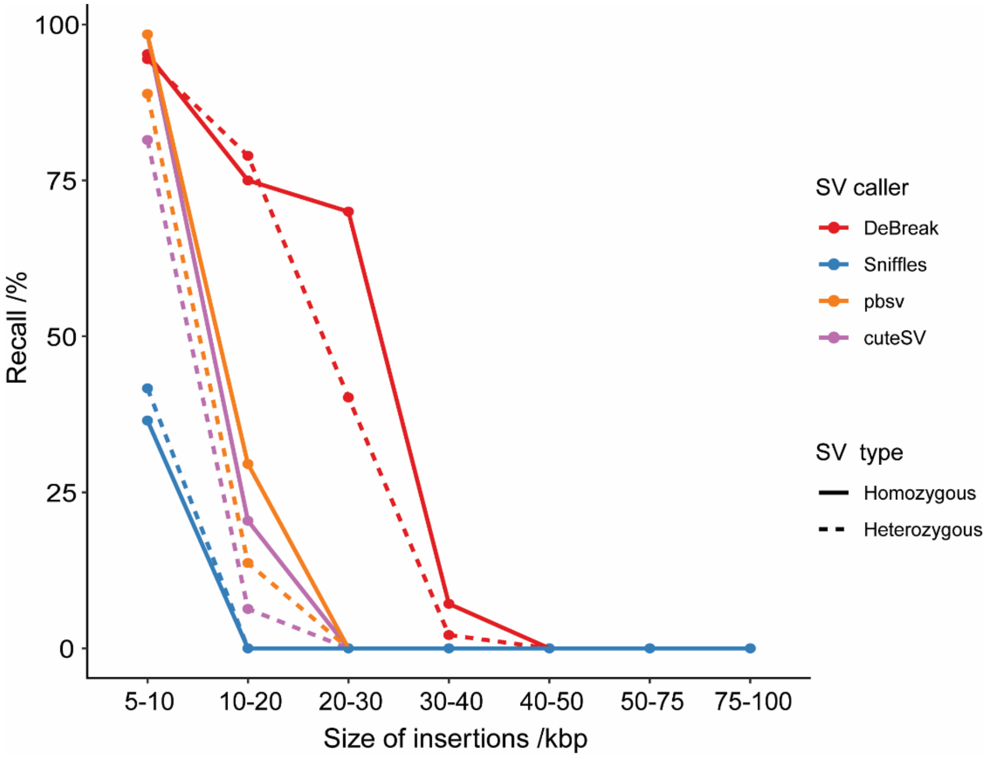

Figure S6 Large insertion detection in simulated datasets. Recall of insertion detection at different size ranges. The average length of sequencing reads was 15kbp. The maximal detectable insertion size is 10kbp for Sniffles, 20kbp for pbsv and cuteSV, and 30kbp for DeBreak.

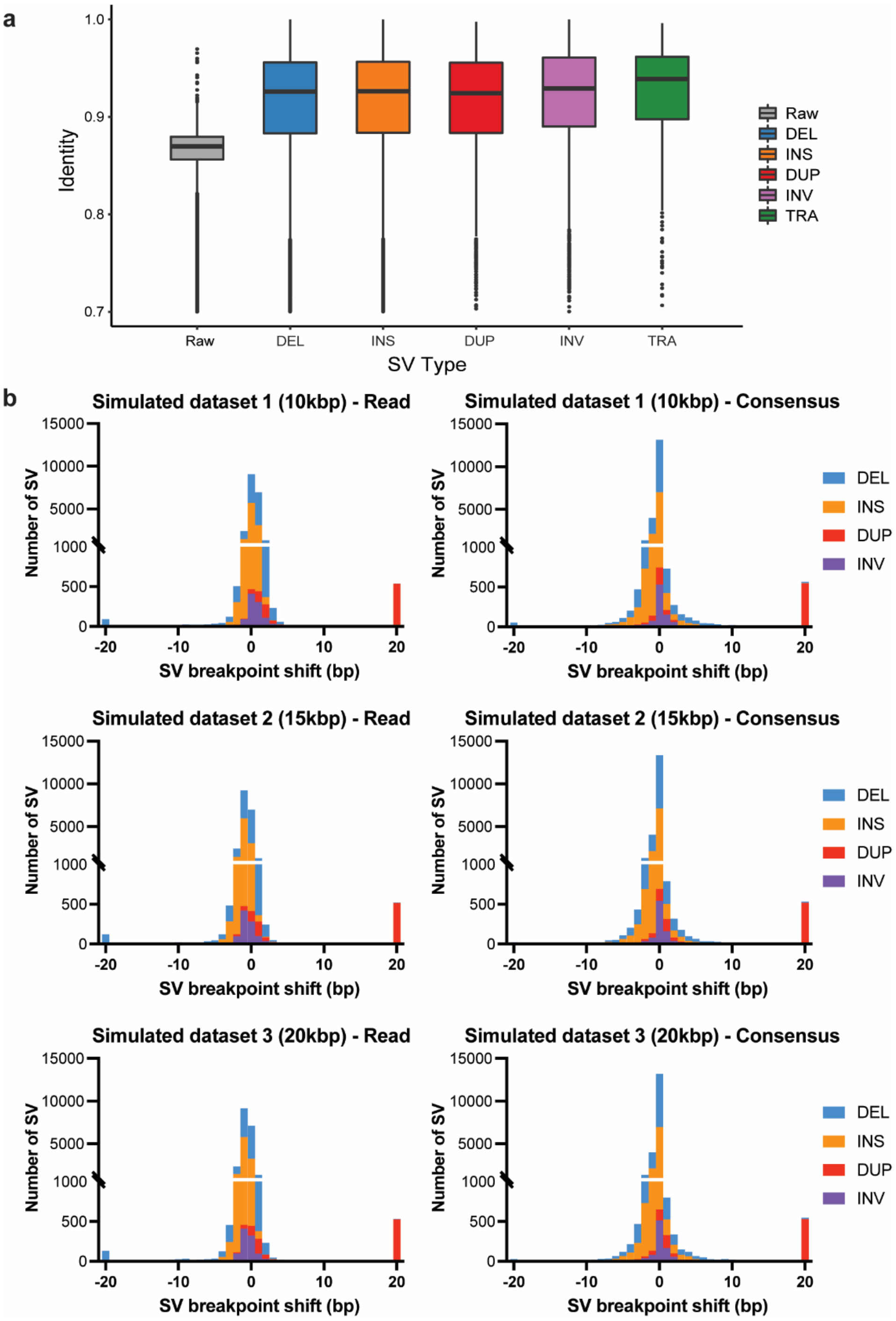

Figure S7 SV breakpoint refinement in simulated datasets. a The identity of reads/consensus sequences compared with sequences around simulated SVs for individual SV type. n=33517 for Raw reads; n=10000 for DEL and INS; n=1000 for DUP and INV; n=200 for TRA. Box plot centerline, median; bounds of box, upper and lower quartiles; whiskers, 1.5*interquartile range; points, outliers. **b** Shift of SV breakpoints inferred from raw reads (left) and from consensus sequences (right) in three simulated datasets (top, center, and bottom). SVs with shifts more than 20bp were combined into the first and last bins.

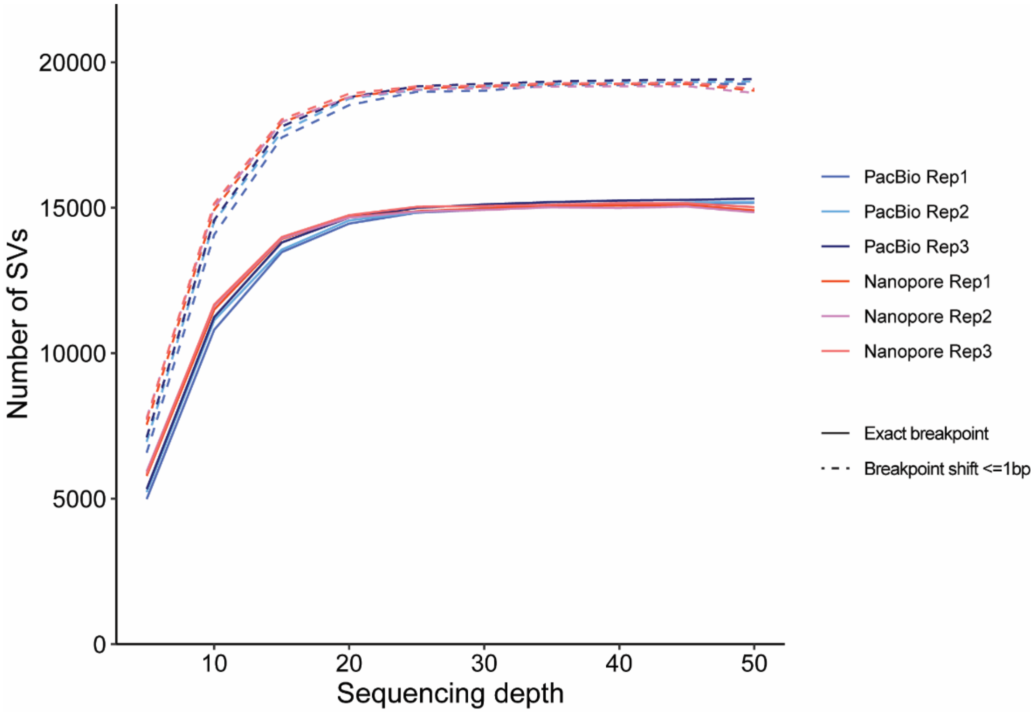

Figure S8 SV breakpoint accuracy in down-sampled simulated datasets. Number of detected SVs with exact breakpoint (solid line) and shift ≤1bp (dashed line) in three PacBio and Nanopore replicates.

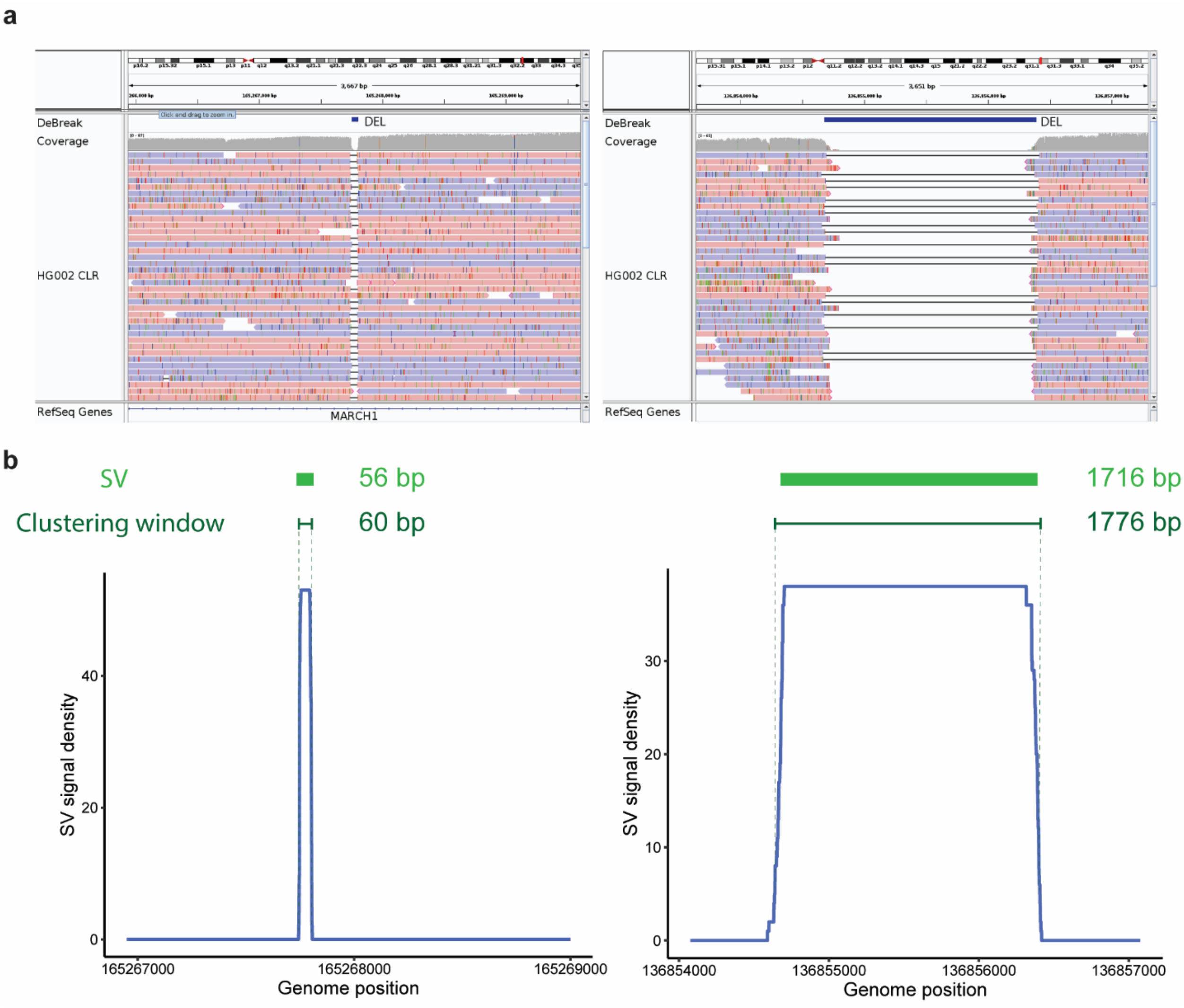

Figure S9 Adjustable clustering window size. a IGV view of read alignments flanking a small SV (left) and a large SV (right). **b** Raw SV signal density of the two SVs shown in **a**. Based on the density pattern of raw SV signals, the clustering window is smaller for shorter SVs (left) and larger for longer SVs (right).

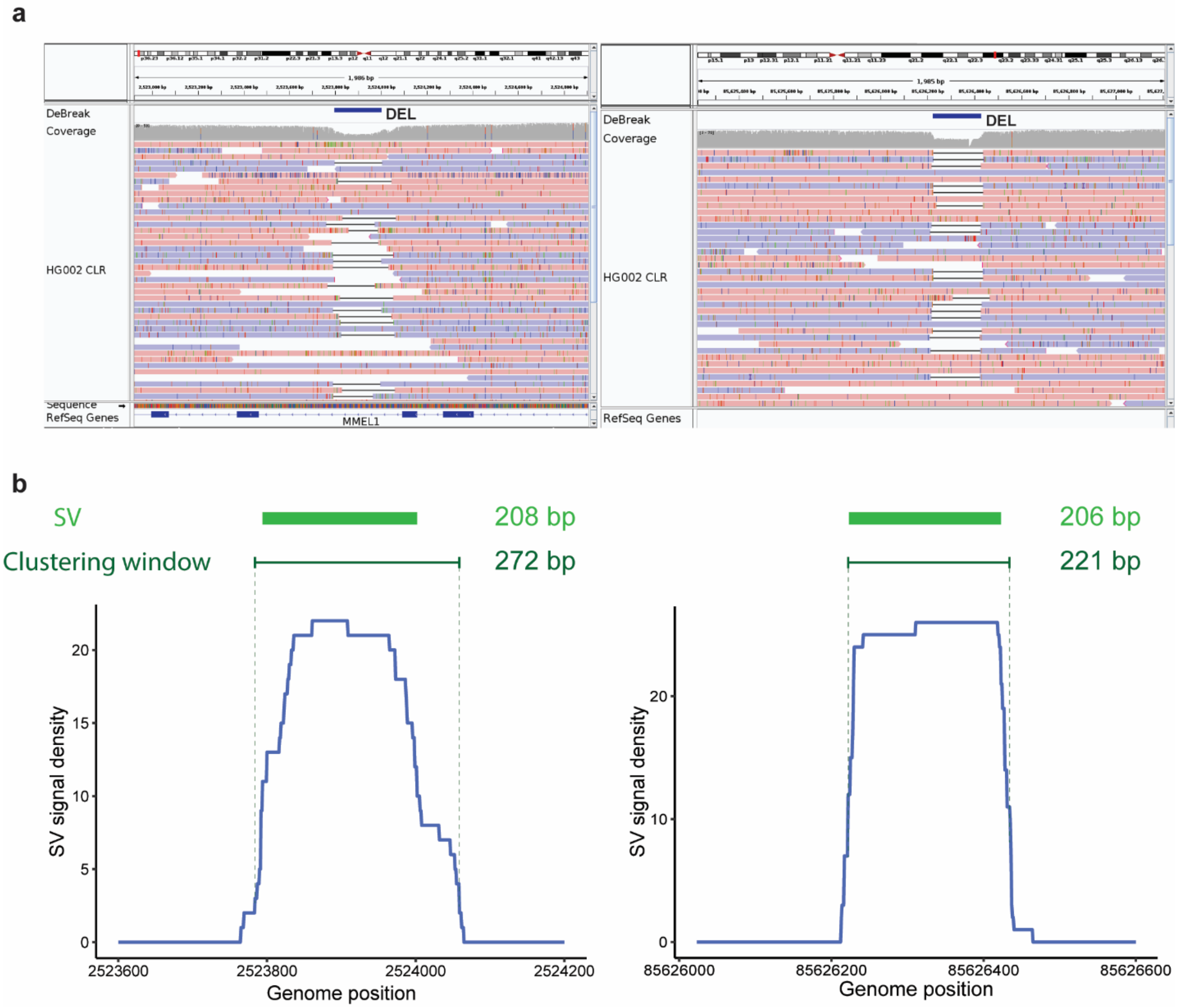

Figure S10 Adjustable clustering window for repeat regions. a IGV view of read alignments flanking SVs located within a low-complexity region (left) and a non-repeat region (right). Raw SV signals have various breakpoint positions in a low-complexity region and relatively consistent breakpoint positions in a non-repeat region. **b** Raw SV signal density of the two SVs shown in **a**. The clustering window size is larger in a low-complexity region when raw signals are diverged (left) and smaller in a non-repeat region (right) to exclude potential noise signals nearby.

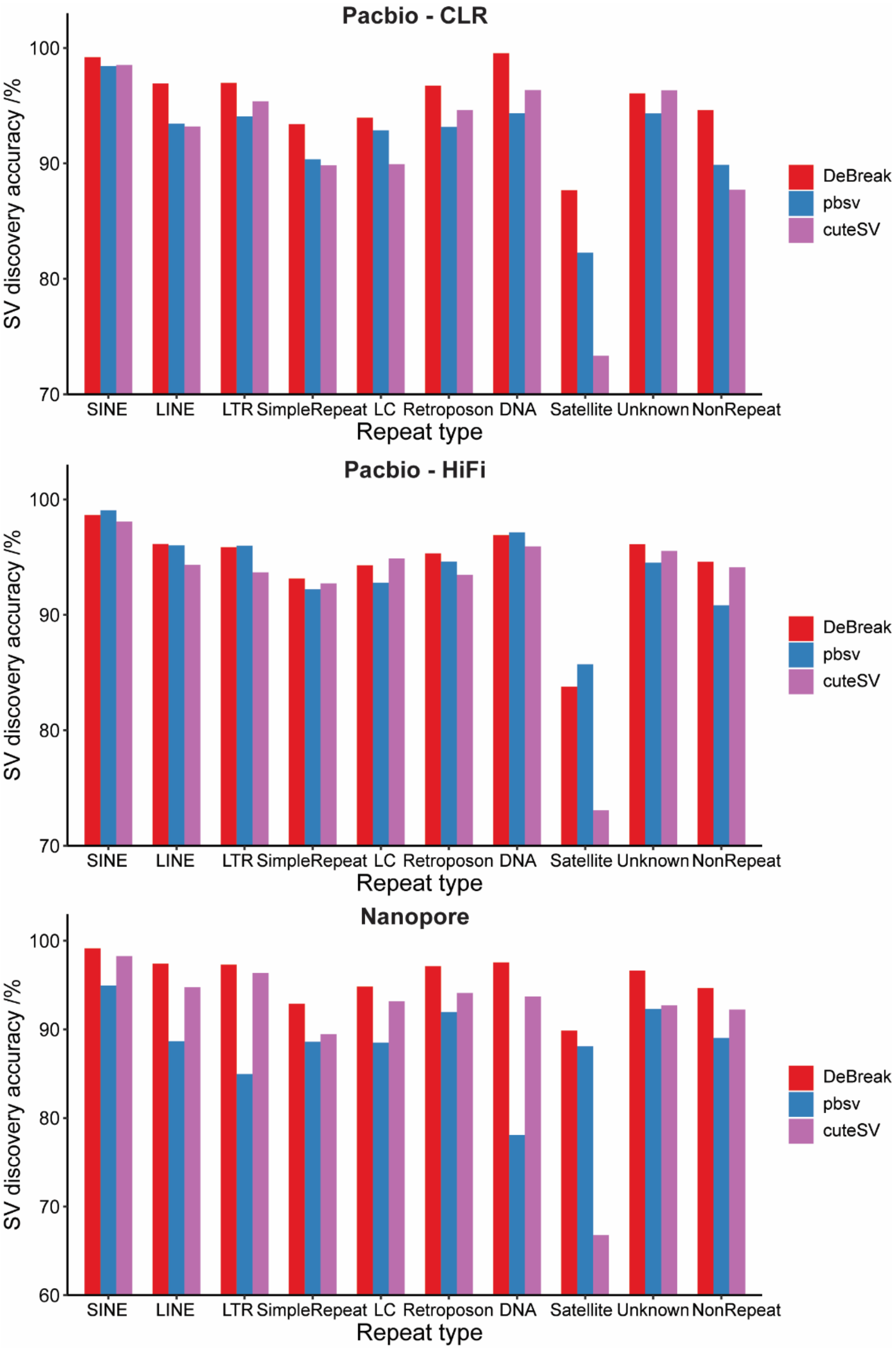

Figure S11 SV discovery accuracy in different repeat types in HG002. F1 score of SV discovery in high-confidence regions of HG002 using PacBio CLR, HiFi and Nanopore data. The repeat type was annotated with RepeatMasker using sequences of longest allele for each SV. LC, low-complexity.

a

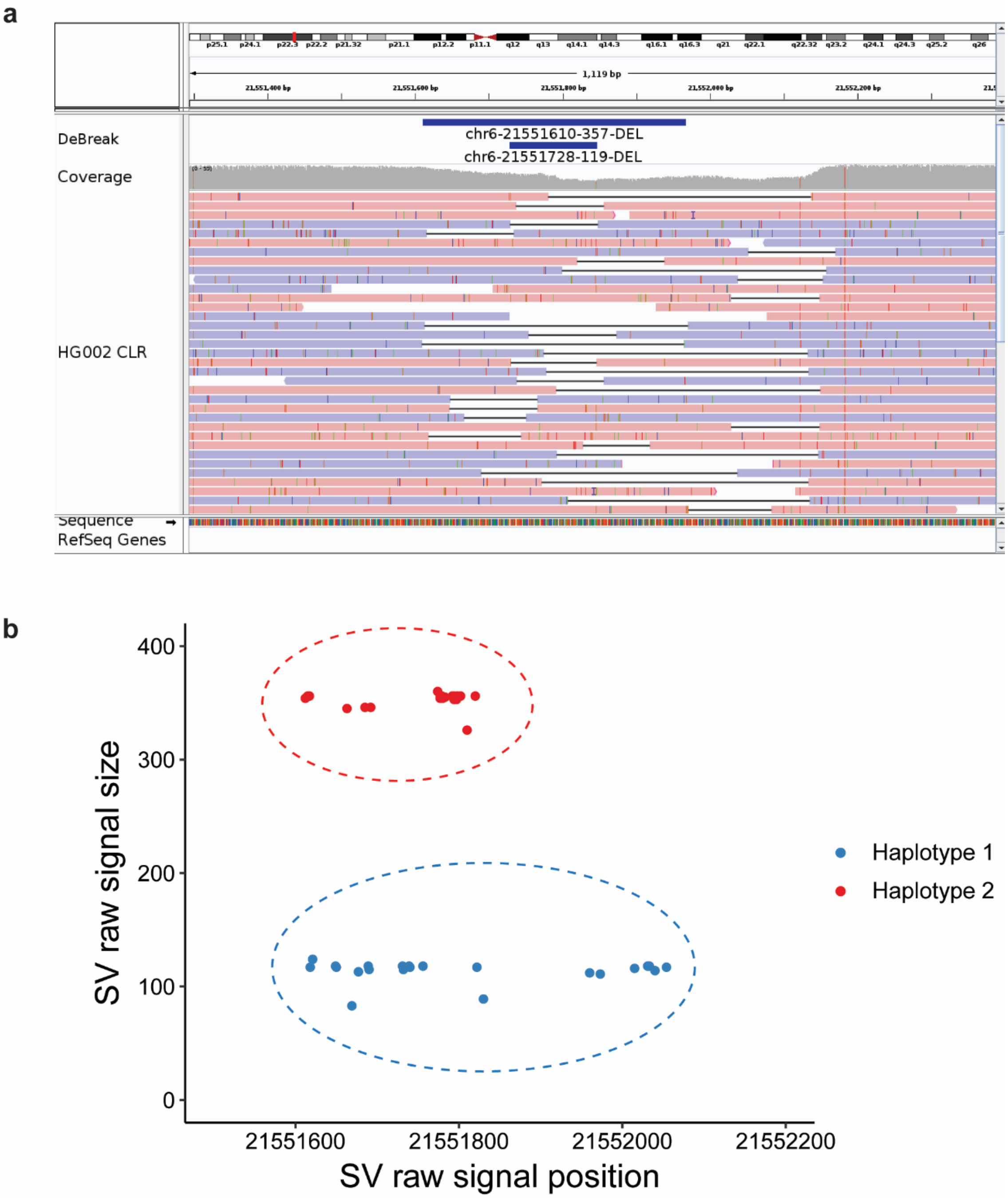

Figure S12 Example of a multi-allele SV in HG002. a IGV view of a multi-allelic SV on chromosome 6. Some reads contain ~100bp (shorter) deletion signals, and other reads contain ~300bp (longer) deletion signals. **b** k-means clustering of SV raw signals from the multi-allelic SV region shown in **a**. Based on SV size and position, SV raw signals are clustered into two groups, each representing one allele.

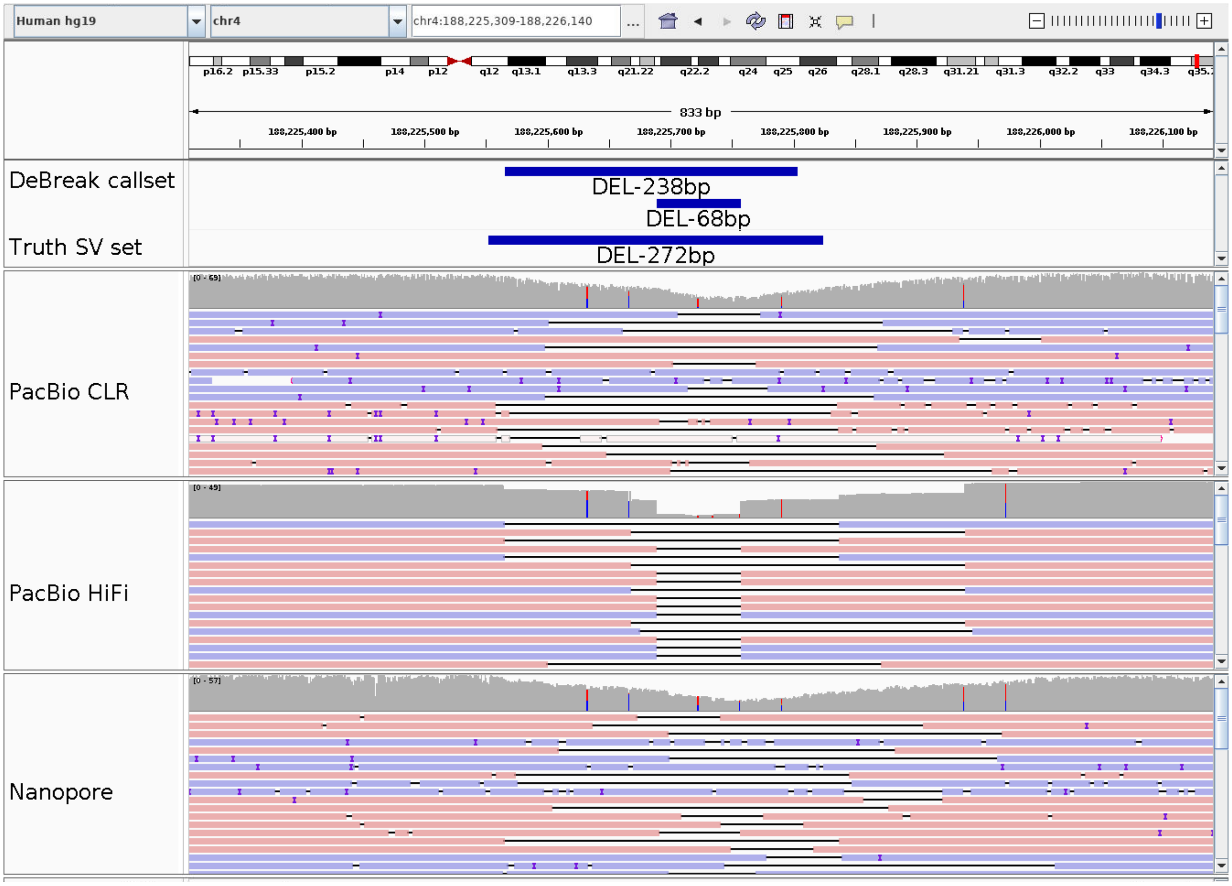

Figure S13 Example mSV in HG002 high-confidence regions. Two alternative alleles were reported by DeBreak (238bp DEL and 68bp DEL) for this mSV, and one of the two alleles matched with the truth SV set (272bp DEL). In PacBio CLR, HiFi, and Nanopore datasets, raw signals of both sizes (~250bp and ~70bp) are present in read alignments in this region.

Table S3 SV genotyping accuracy in HG002

	PacBio CLR			PacBio HiFi			Nanopore		
	DEL	INS	Total	DEL	INS	Total	DEL	INS	Total
**DeBreak**	**93.22**	**84.00**	**87.98**	90.46	84.70	87.21	**92.18**	86.38	**88.92**
**Sniffles**	47.47	38.51	42.43	52.38	45.77	48.70	61.69	56.08	58.55
**pbsv**	92.35	75.09	82.14	93.26	75.19	82.34	80.22	79.81	79.99
**cuteSV**	92.53	78.99	84.52	**91.95**	**88.56**	**90.02**	88.05	**87.45**	87.71

The genotyping accuracy is calculated as the number of SVs with the correct genotype divided by the total number of SVs reported by each SV caller. The highest genotyping accuracy in each group is shown in bold. The unit of genotyping accuracy is %.

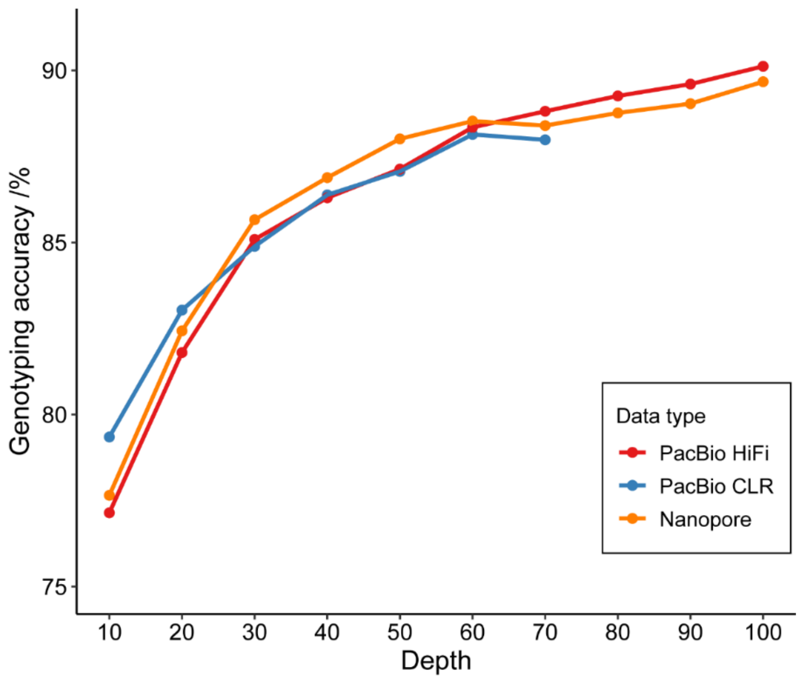

Figure S14 Genotyping accuracy in down-sampled datasets in HG002. The PacBio CLR dataset was downsampled from 10x to 70x. The PacBio HiFi and Nanopore datasets were downsampled from 10x to 100x.

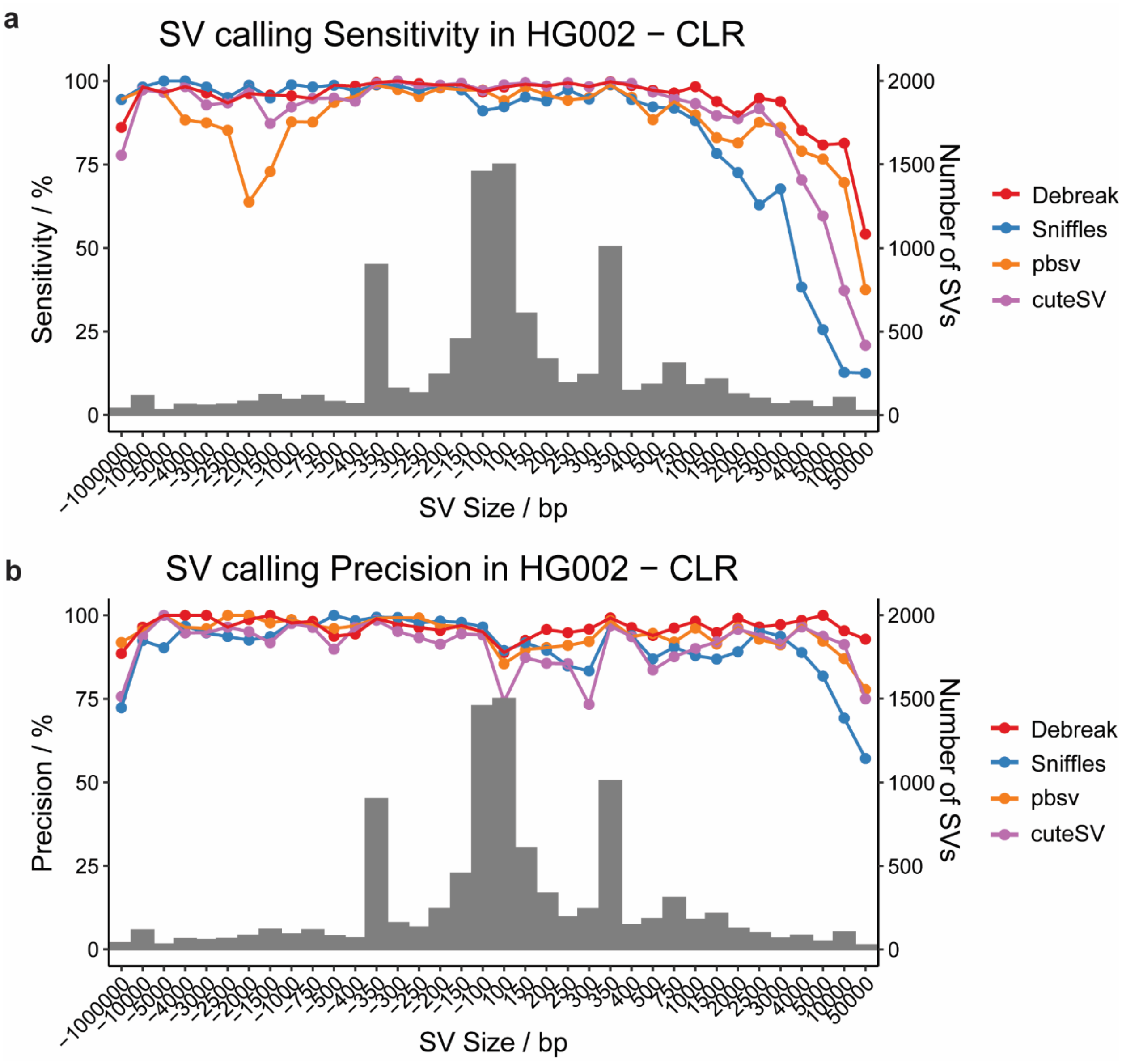

Figure S15 SV calling recall and precision for insertions (positive SV size) and deletions (negative SV size) for four tested SV callers in HG002 high-confidence regions. The bar plot indicates the number of SVs in each size range.

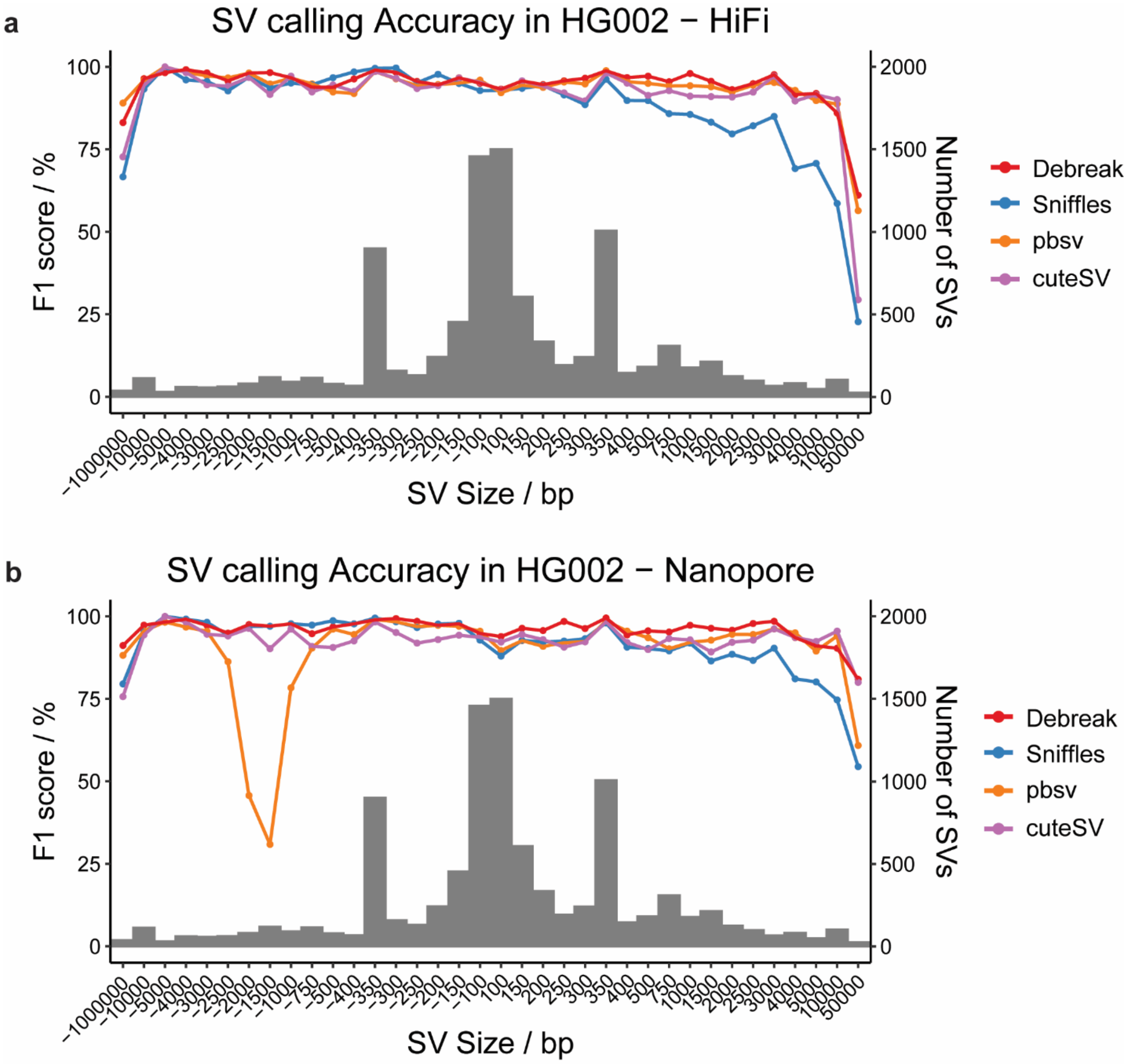

Figure S16 SV calling accuracy for insertions (positive SV size) and deletions (negative SV size) for four tested SV callers in PacBio HiFi and Nanopore datasets. The bar plot indicates the number of SVs in each size range.

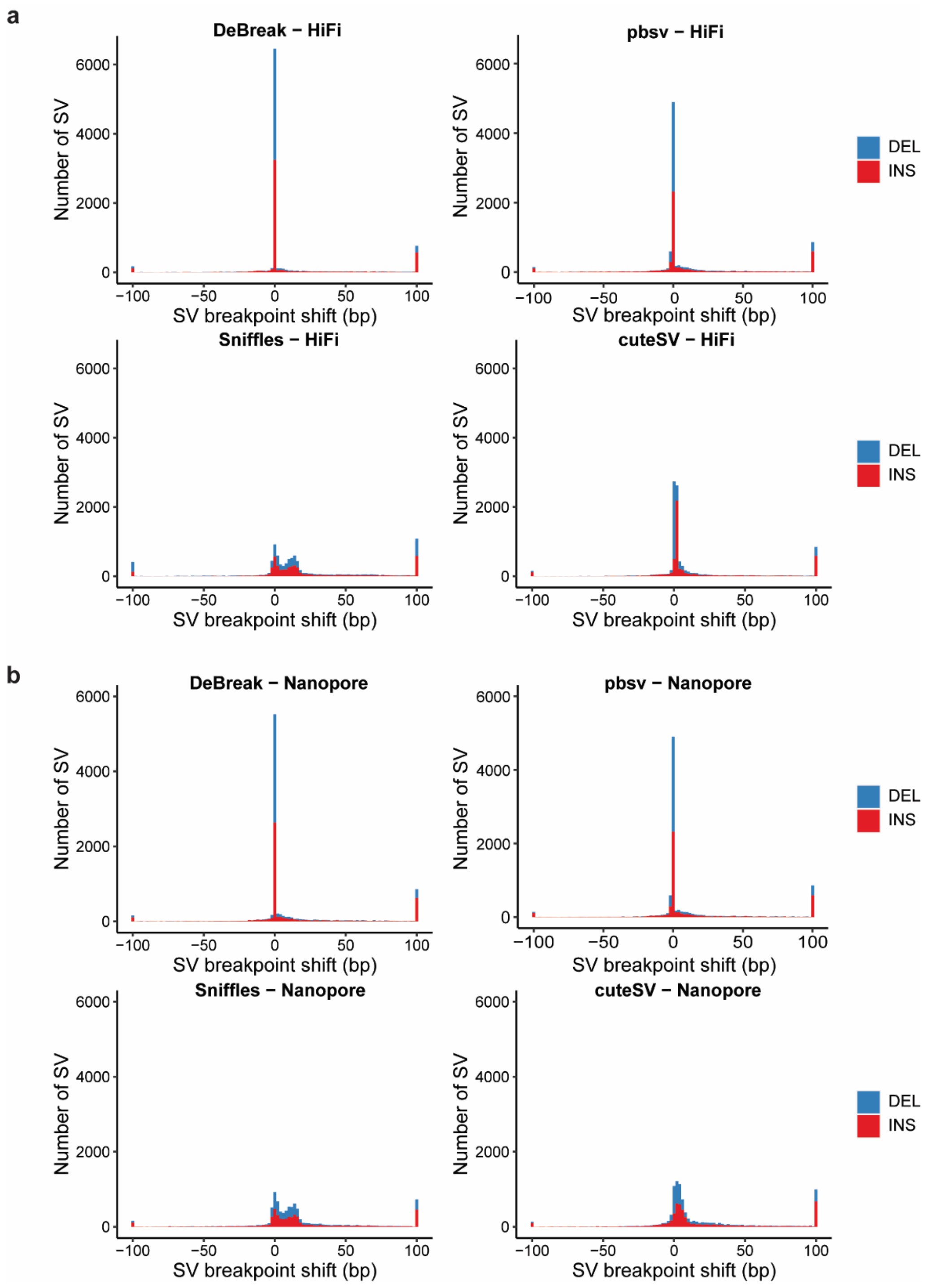

Figure S17 SV breakpoint accuracy in HG002 HiFi and Nanopore datasets. a SV breakpoint shift of four SV callers in the HiFi dataset. 64%, 57%, 5%, and 25% of SVs were identified with exact breakpoint position by DeBreak, pbsv, Sniffles, and cuteSV, respectively. **b** SV breakpoint shift of four SV callers in Nanopore dataset. 54%, 49%, 5%, and 7% of SVs were identified with exact breakpoint position by DeBreak, pbsv, Sniffles and cuteSV, respectively.

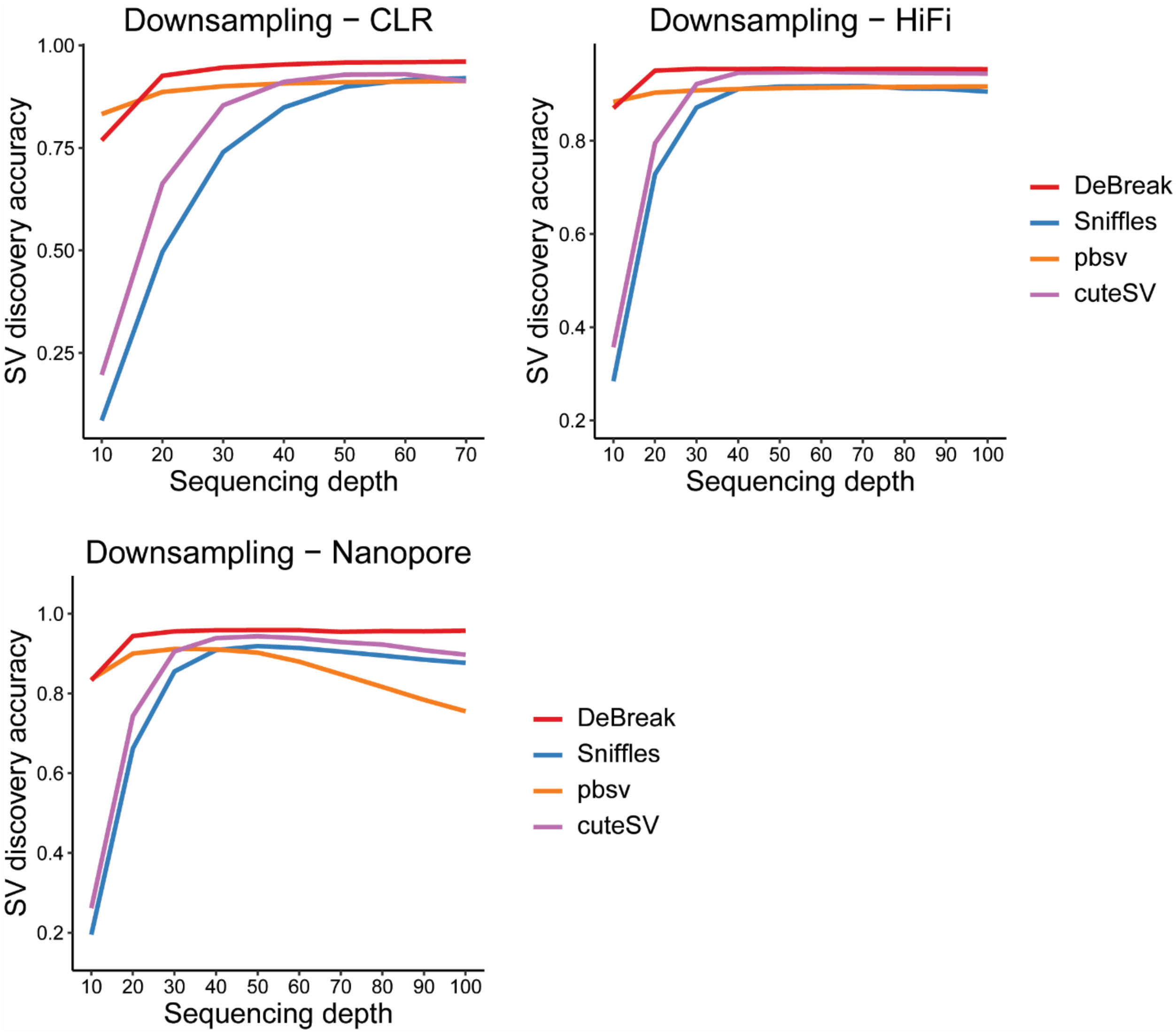

Figure S18 SV discovery accuracy in down-sampled datasets under default settings. Sniffles and cuteSV demonstrate lower accuracy at lower sequencing depths.

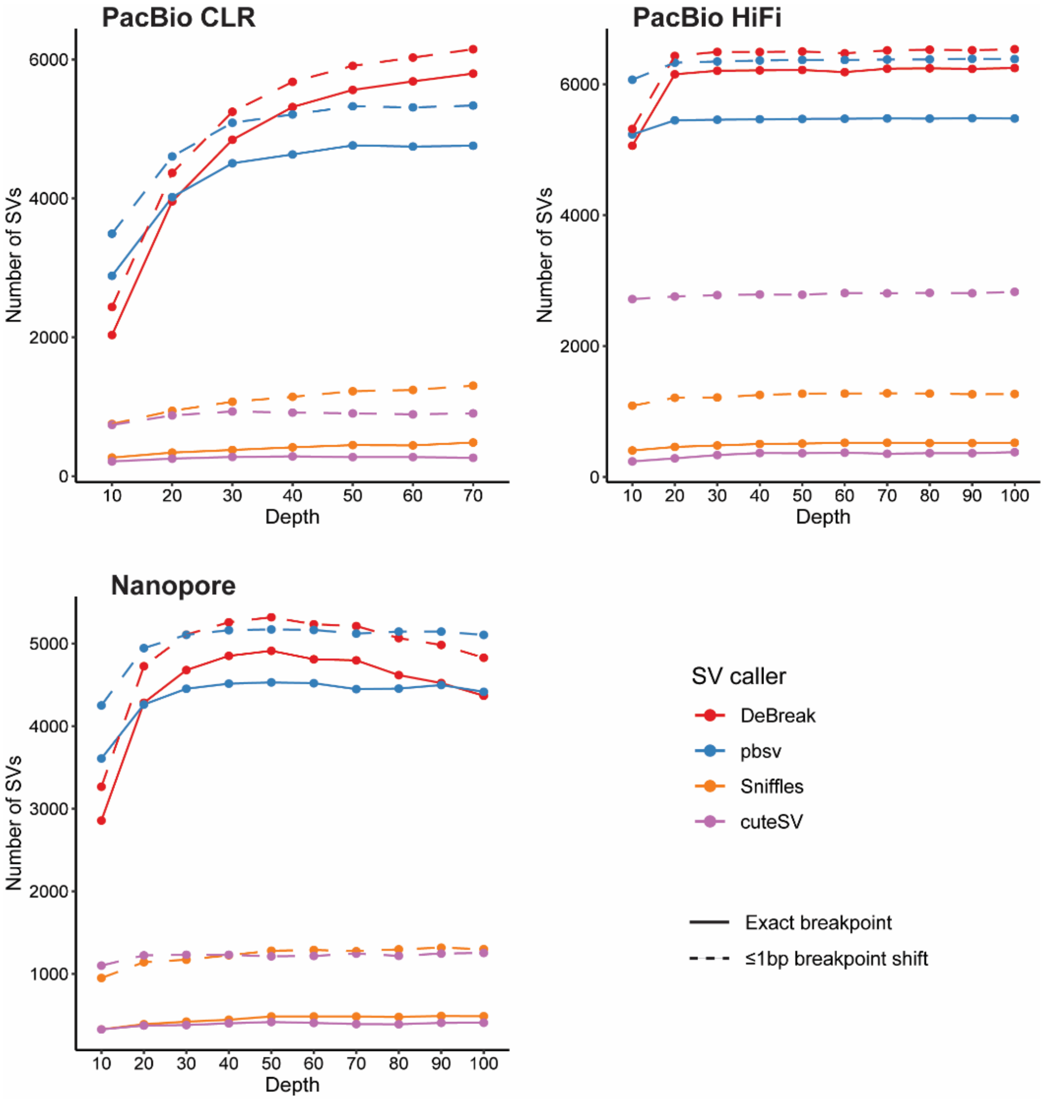

Figure S19 Breakpoint accuracy in downsampled datasets in HG002. Number of SV detected with exact breakpoint and with ≤1bp shift in breakpoints using PacBio CLR, HiFi, and Nanopore data at different sequencing depths. PacBio CLR data was downsampled from 10x to 70x. PacBio HiFi and Nanopore data were downsampled from 10x to 100x.

Table S4 SV discovery accuracy compared to assembly-based SV callsets

	DeBreak			pbsv			cuteSV			Sniffles		
**Sample**	**DEL**	**INS**	**Total**	**DEL**	**INS**	**Total**	**DEL**	**INS**	**Total**	**DEL**	**INS**	**Total**
**CLR**												
HG00096	80.52	78.59	**79.34**	76.51	69.85	72.59	78.72	77.73	78.12	78.39	69.54	73.18
HG01505	80.77	78.72	**79.52**	76.96	69.51	72.57	79.18	77.51	78.16	78.03	69.00	72.73
HG01596	80.15	75.83	**77.45**	75.46	68.72	71.47	78.28	72.10	74.39	76.76	66.80	70.69
**HiFi**												
HG02818	81.90	80.57	**81.12**	78.43	69.05	73.19	78.45	77.08	77.65	77.75	65.73	71.01
HG03486	82.17	81.58	**81.82**	78.56	69.30	73.40	79.71	78.65	79.09	79.14	68.22	73.01
NA12878	80.78	81.66	**81.31**	77.80	69.17	72.87	77.32	76.54	76.85	73.64	63.88	68.00

SV discovery accuracy was evaluated with the assembly-based SV callset as the ground truth. The highest accuracy (F1 score) among four tested alignment-based SV callers is shown in bold in each sample. The unit of F1 score is %.

Table S5. SV discovery recall and precision compared to assembly-based SV callsets

	DeBreak				pbsv				cuteSV				Sniffles			
	R-D	P-D	R-I	P-I	R-D	P-D	R-I	P-I	R-D	P-D	R-I	P-I	R-D	P-D	R-I	P-I
**CLR**																
HG00096	79.46	81.62	78.08	79.11	77.24	80.25	75.64	79.94	78.44	74.67	65.40	74.95	75.27	81.29	61.67	80.79
HG01505	79.40	82.20	76.68	80.87	77.41	81.04	74.47	80.81	78.99	75.03	64.51	75.34	75.04	81.04	59.80	82.42
HG01596	77.68	82.79	75.63	76.03	76.04	80.66	73.89	70.41	77.44	73.58	63.59	74.75	71.21	84.47	59.53	76.14
Total	**78.85**	**82.19**	**76.79**	78.64	76.90	80.65	74.66	76.79	78.30	74.43	64.50	75.01	73.86	82.18	60.33	**79.71**
**HiFi**																
HG02818	81.44	82.36	76.54	85.04	78.40	78.50	73.41	81.13	80.36	76.59	60.19	80.97	75.94	80.18	58.66	87.12
HG03486	83.14	81.22	79.31	83.98	81.29	78.20	77.26	80.09	81.71	75.64	60.85	80.47	78.20	79.18	60.94	86.54
NA12878	81.80	79.80	78.58	84.99	75.91	78.78	72.78	80.71	79.19	76.45	59.90	81.81	72.65	78.30	57.98	87.07
Total	**82.15**	**81.19**	**78.13**	84.65	78.71	78.46	74.57	80.62	80.50	76.21	60.33	81.04	75.79	79.28	59.26	**86.90**

SV discovery accuracy was evaluated with the assembly-based SV callset as the ground truth. The highest recall and precision among four tested alignment-based SV callers is marked in bold. The unit of recall and precision is %.
R-D, recall for deletion. P-D, precision for deletion. R-I, recall for insertion. P-I, precision for insertion.

Table S6 SV genotyping accuracy in HGSVC samples

	DeBreak	pbsv	cuteSV	Sniffles
**CLR**				
HG00096	72.70	67.81	**75.06**	45.88
HG01505	73.18	67.82	**75.82**	46.13
HG01596	**70.04**	65.11	68.75	39.05
**HiFi**				
HG02818	72.62	72.91	**74.91**	45.45
HG03486	71.75	72.54	**74.13**	45.25
NA12878	71.68	70.43	**73.52**	39.06

SV genotyping accuracy was assessed with the assembly-based SV callset as the ground truth. The highest accuracy among four tested alignment-based SV callers is shown in bold for each sample. The unit of genotyping accuracy is %.

Table S7 DeBreak mSV discovery in HGSVC samples

	mSV	Alternative allele	Validation rate	mCNV
**CLR**				
HG00096	1097	2194	73.38	12
HG01505	1011	2022	71.96	4
HG01596	992	1984	70.46	7
Total	3100	6200	71.98	23
**HiFi**				
HG02818	1031	2062	73.81	7
HG03486	1187	2374	72.11	10
NA12878	879	1758	67.86	7
Total	3097	6194	71.47	24

Alternative alleles that are also reported in assembly-based SV callset were considered as validated. mCNV is classified using k-mer counts. The unit of validation rate is %.

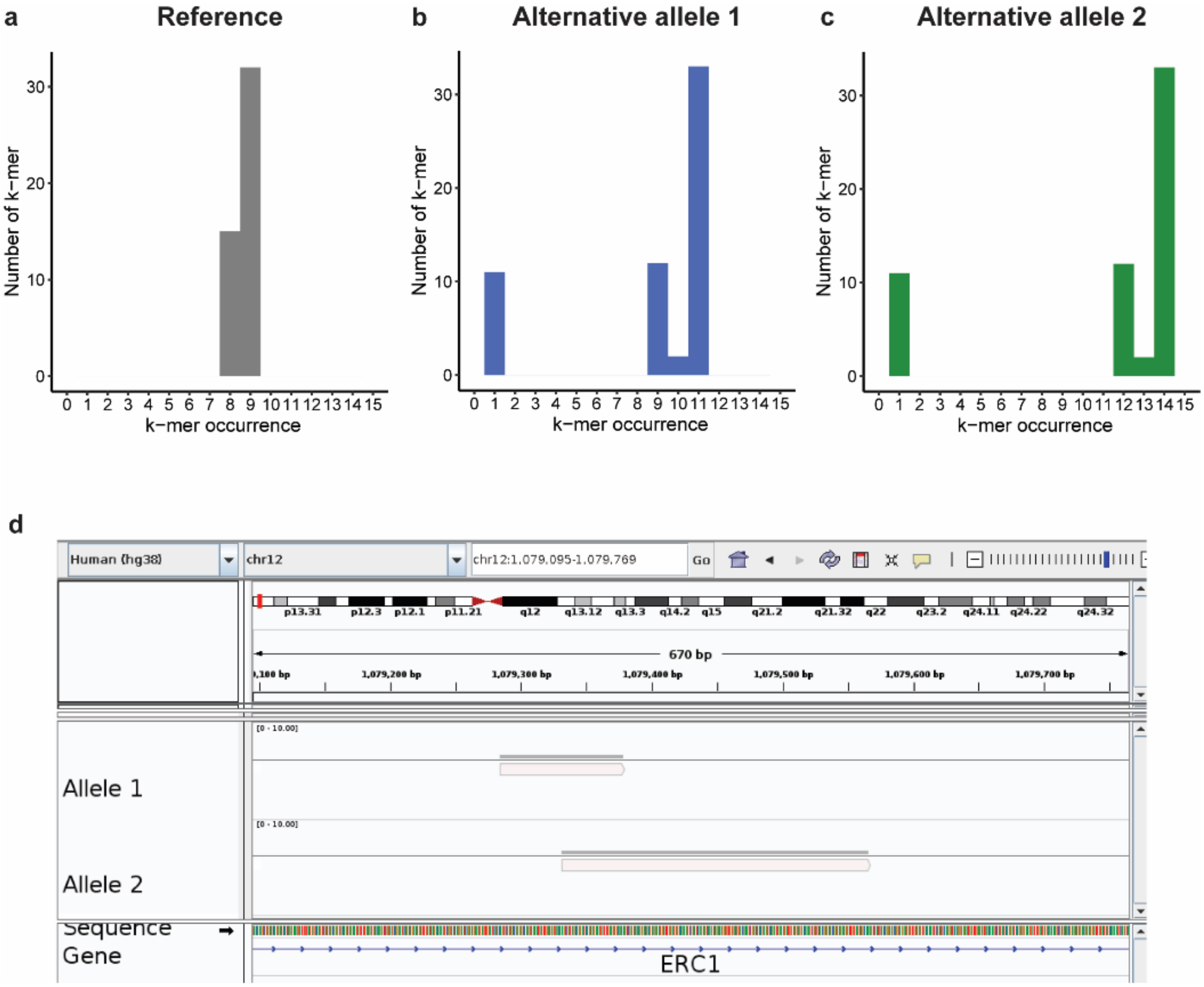

Figure S20 Example an mCNV event (chr12-1079285-94bp-INS/chr12-1079285-235bp-INS) in HG02818. a k-mer occurrence of reference sequences. A peak of k-mer at 9 indicates 9 copies of a specific repeat unit in the reference genome. **b, c** k-mer occurrence of alternative allele 1 (**b**, INS of 94bp) and alternative allele 2 (**c**, INS of 235bp). 11 and 14 copies of the repeat unit are present in the two alleles. **d** Alignment of inserted sequences of two alternative alleles. Both alleles can be fully aligned to the reference genome near the mSV breakpoint.

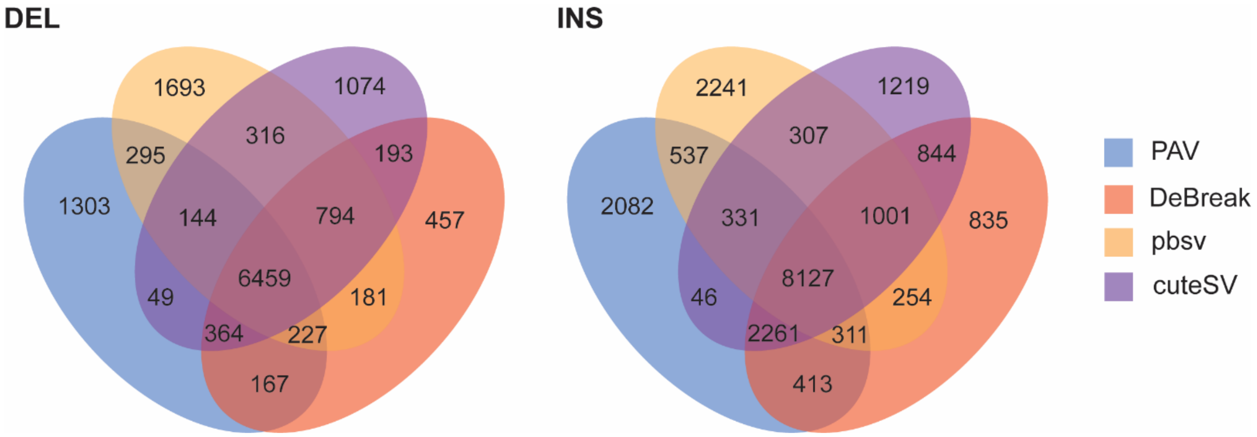

Figure S21 SV discovery consistency between alignment-based and assembly-based SV discovery approaches. Venn diagrams showing the overlap between alignment-based and assembly-based SV callsets. Numbers indicate the number of SVs in each group.

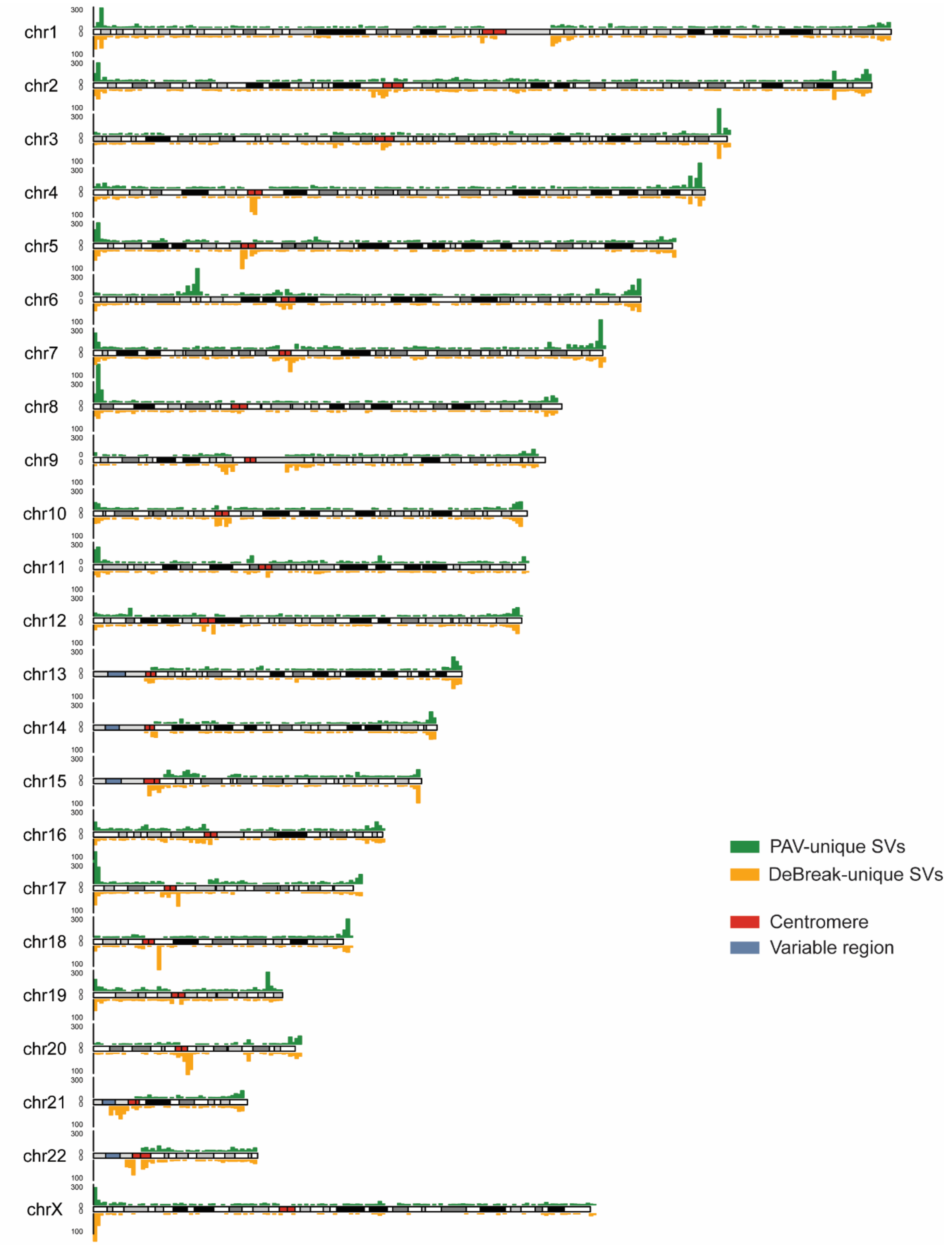

Figure S22 PAV-unique and DeBreak-unique SV distribution on the genome. The PAV-unique SVs (green) are enriched at the telomere regions. DeBreak-unique SVs (orange) are enriched at the centromere and telomere regions.

Table S8 SV discovery accuracy in CHM13

	Deletion			Insertion			Total		
	Recall	Precision	F1	Recall	Precision	F1	Recall	Precision	F1
**CLR**									
DeBreak	82.41	88.58	**85.38**	79.29	87.19	83.05	80.45	87.72	**83.93**
Sniffles	78.66	86.01	82.17	63.03	90.42	74.28	68.86	88.49	77.45
pbsv	81.84	84.76	83.27	70.74	85.52	77.43	74.88	85.21	79.71
cuteSV	83.84	86.35	85.08	81.44	84.74	**83.06**	82.33	85.35	83.81
**HiFi**									
DeBreak	83.71	86.12	**84.90**	80.81	89.89	**85.11**	81.89	88.39	**85.02**
Sniffles	79.44	83.84	81.58	60.42	88.75	71.89	67.51	86.50	75.84
pbsv	80.69	83.18	81.92	65.04	88.26	74.89	70.88	86.01	77.71
cuteSV	85.93	79.68	82.69	84.03	85.07	84.55	84.74	82.97	83.84
**Nanopore**									
DeBreak	85.72	82.83	**84.25**	83.46	87.70	**85.53**	84.30	85.76	**85.03**
Sniffles	81.87	78.57	80.19	64.49	88.54	74.63	70.97	83.94	76.91
pbsv	84.44	54.55	66.28	68.12	86.07	76.05	74.21	69.06	71.54
cuteSV	88.52	71.71	79.23	86.09	83.24	84.64	87.00	78.41	82.48

SV discovery accuracy was evaluated with the assembly-based SV callset as the ground truth. The highest F1 score in each SV type are shown in bold. The unit of recall, precision, and F1 score is %.

Table S9 SV genotyping accuracy in CHM13

	CLR	HiFi	Nanopore
DeBreak	77.00	**86.02**	**78.74**
Sniffles	32.03	26.19	48.73
pbsv	62.65	81.31	59.33
cuteSV	**77.33**	74.36	61.50

SV genotyping accuracy was evaluated with only ‘GT=1/1’ as correct genotype. The highest genotyping accuracy in each data type are shown in bold. The unit of genotyping accuracy is %.

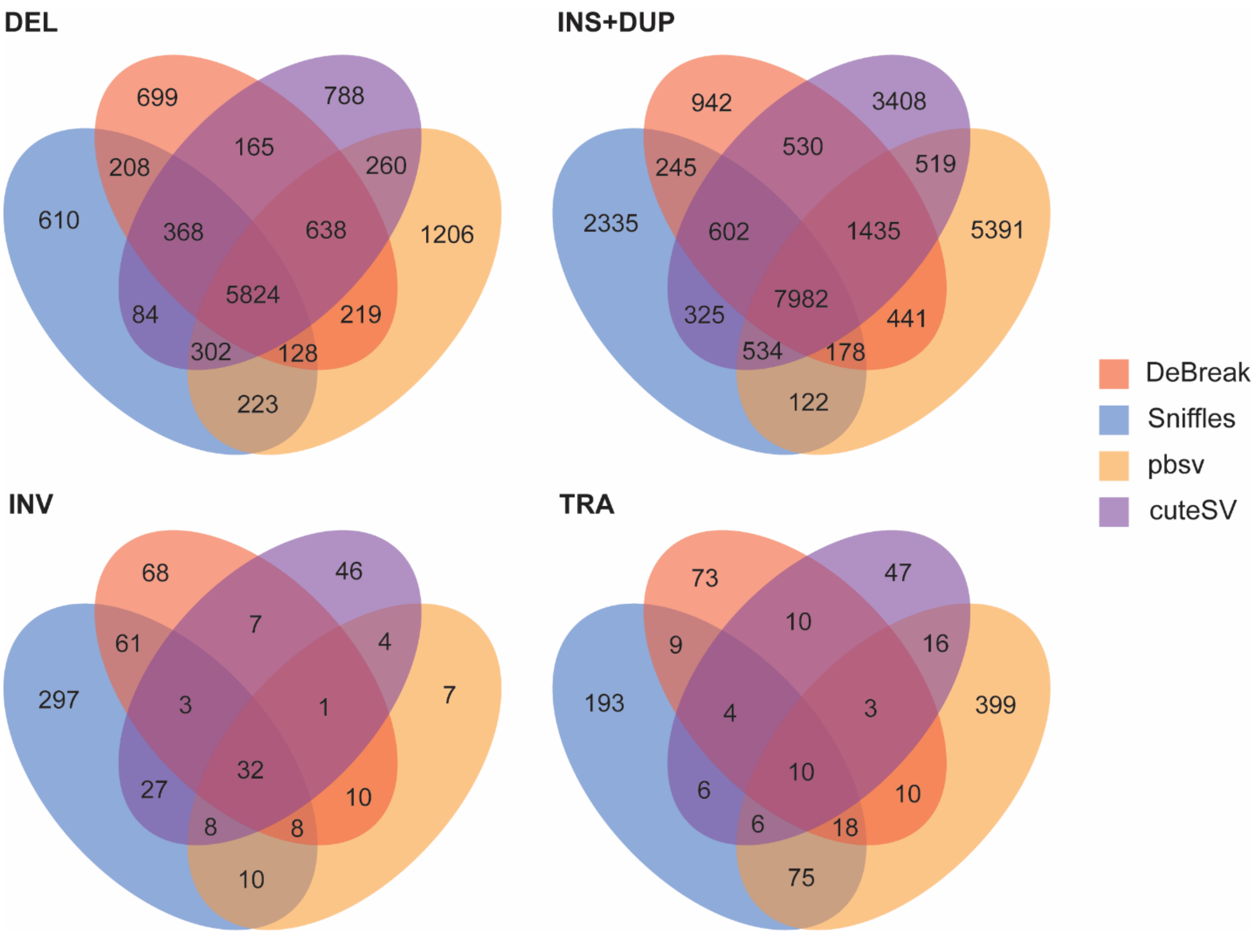

Figure S23 SV discovery in SKBR3 cell line. Venn diagrams showing the overlap between SV callsets from four SV callers. Insertions and duplications have been merged for comparison, as duplications are sometimes considered as insertions.

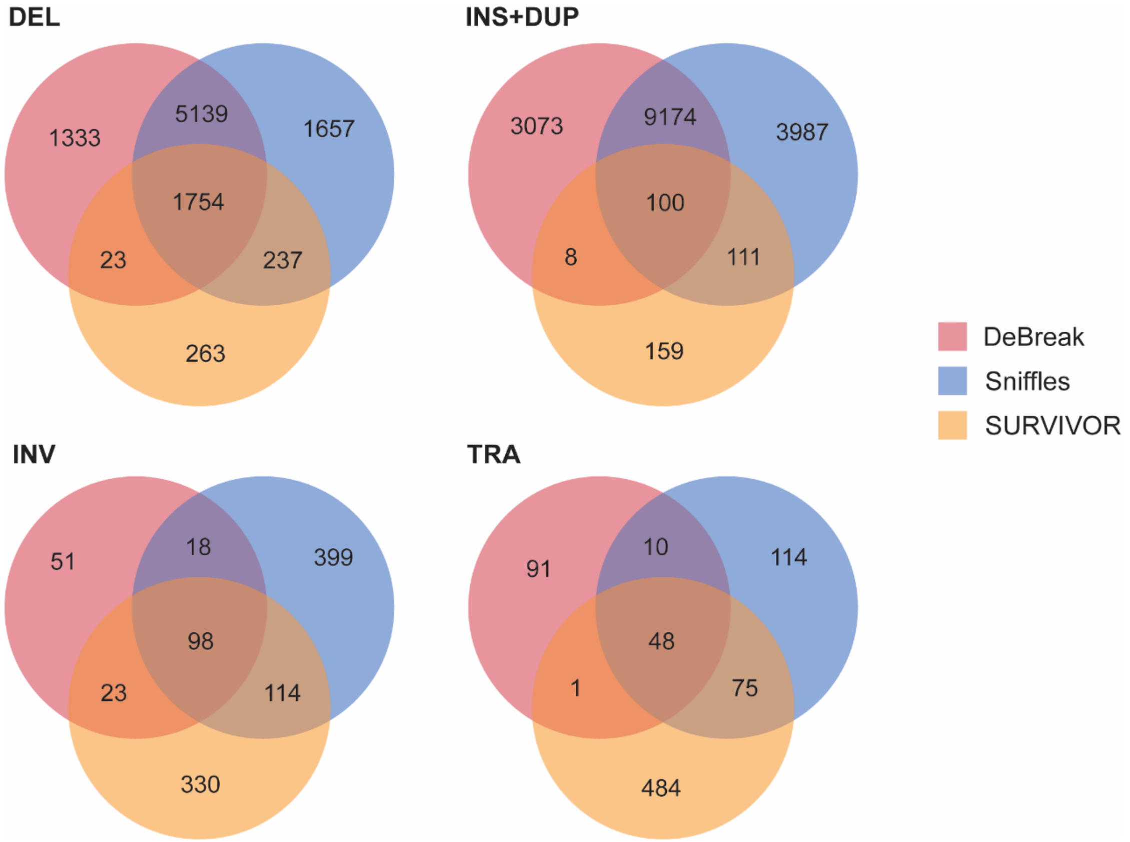

Figure S24 SV discovery in SKBR3 cell line. Venn diagrams showing the overlap between DeBreak SV callset and SV calls previously reported from short-read (SURVIVOR) and long-read data (Sniffles). Numbers indicate the number of SVs in each category.

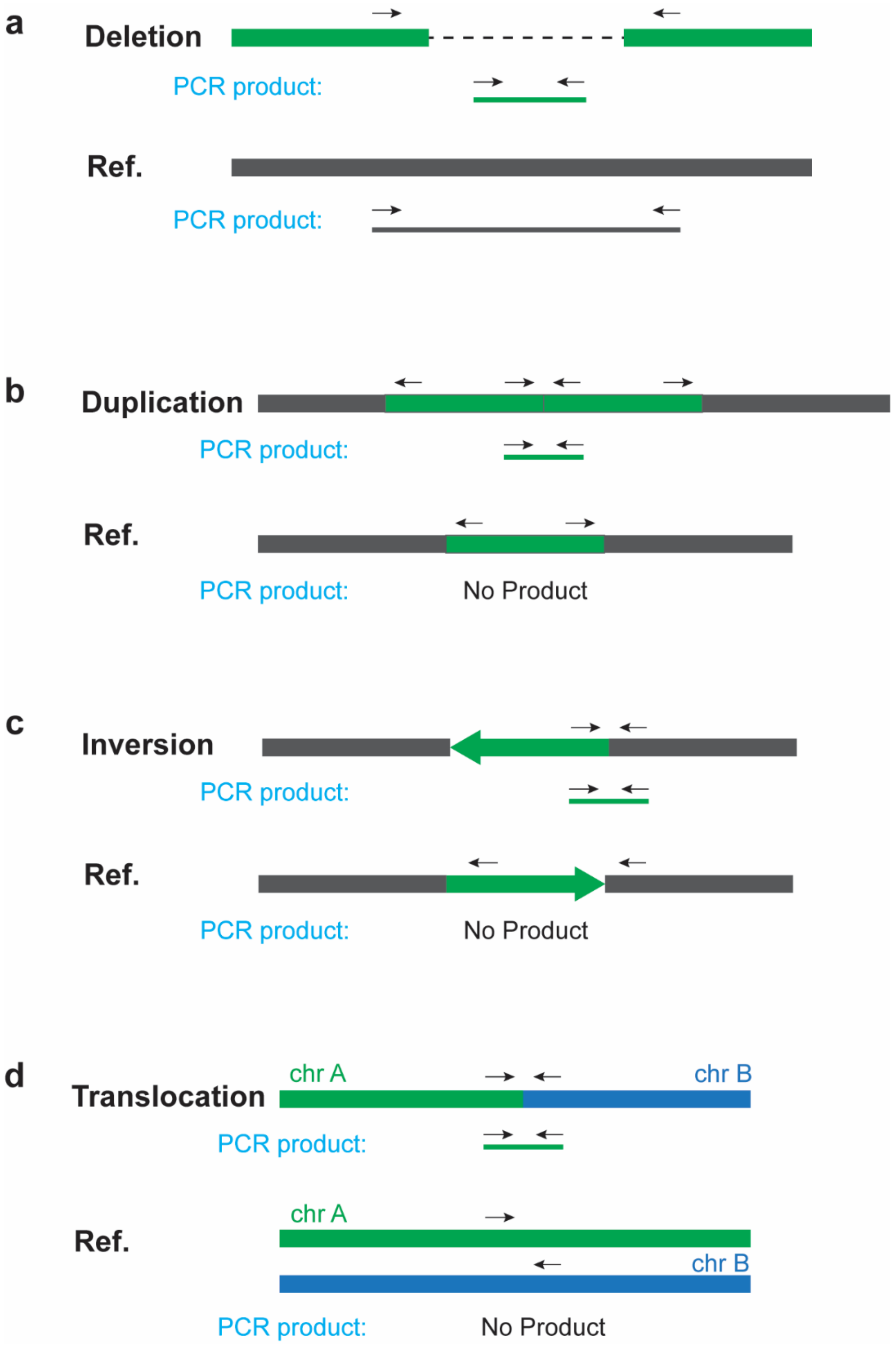

Figure S25 PCR primer for validation. PCR primer design for deletions (**a**), duplications (**b**), inversions (**c**), and translocations (**d**). For deletions, the PCR product size is much smaller when the SV is a true event than for false positive (reference allele). For duplications, inversions, and translocations, the PCR product is expected only when SV is a true event.

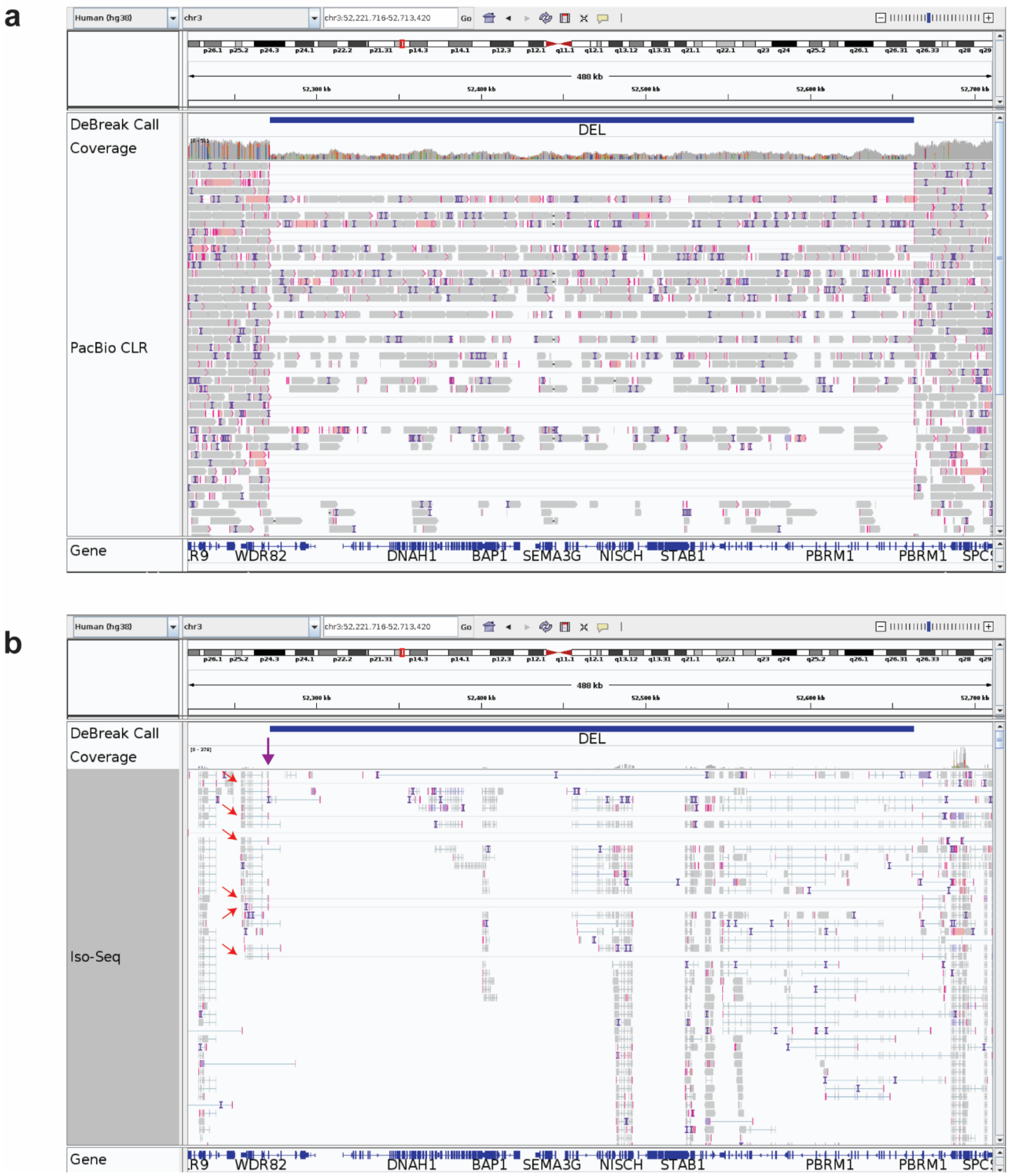

Figure S26 Gene fusion of WDR82 and PBRM1. IGV view of PacBio CLR (**a**) and Iso-Seq data (**b**) at the gene fusion junction. The 'DeBreak Call' panel shows SVs identified from PacBio data with DeBreak. Red arrows indicate IsoSeq reads that contain sequences from both WDR82 and PBRM1. The purple arrow indicates the gene fusion junction position inferred from IsoSeq reads.

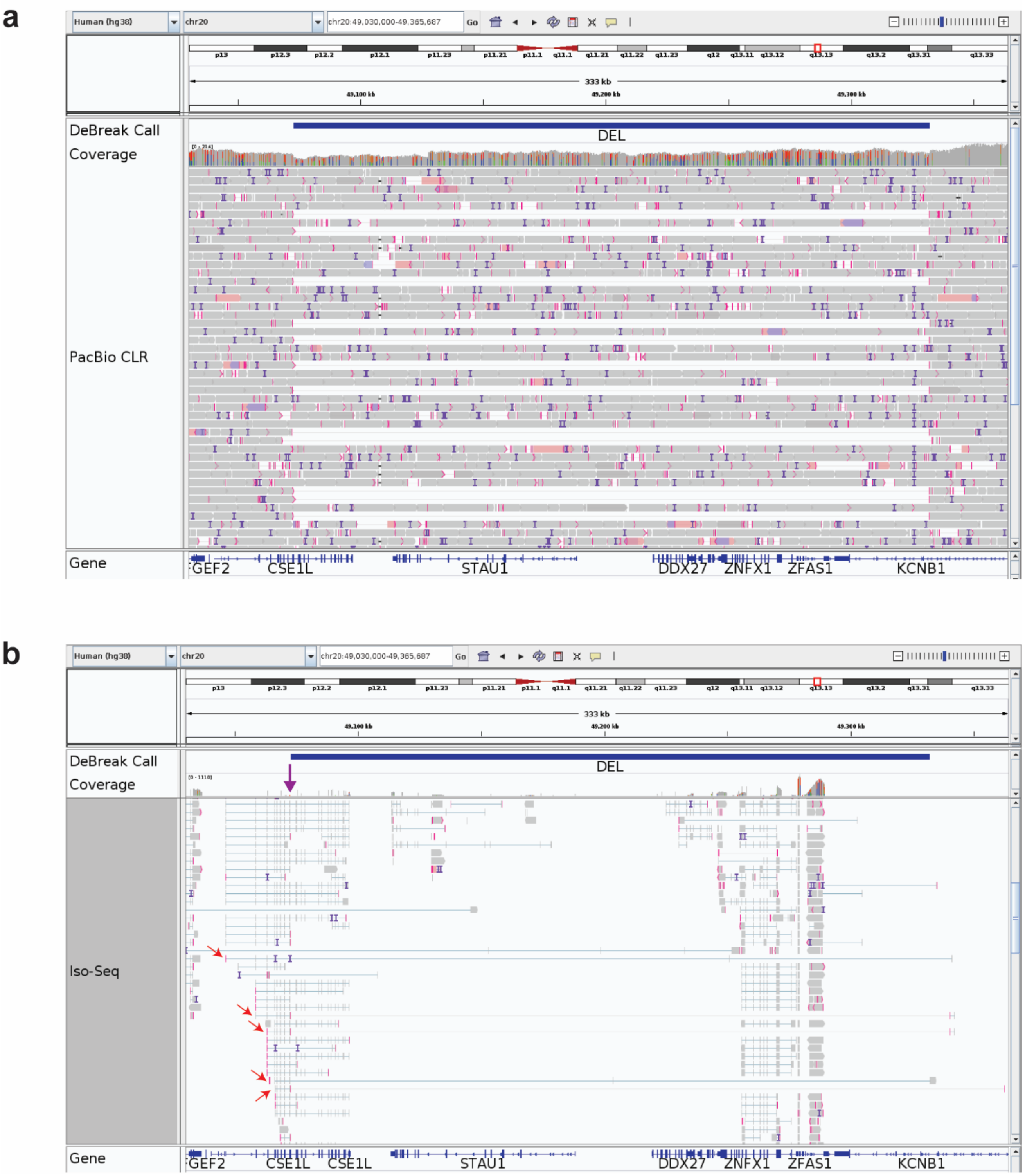

Figure S27 Gene fusion of CSE1L and KCNB1. IGV view of PacBio CLR (**a**) and Iso-Seq data (**b**) at the gene fusion junction. The 'DeBreak Call' panel shows SVs identified from PacBio data with DeBreak. Red arrows indicate the IsoSeq reads that contain sequences from both CSE1L and KCNB1. The purple arrow indicates the gene fusion junction position inferred from IsoSeq reads.

Table S10 Runtime and memory usage of SV callers

	DeBreak	Sniffles	pbsv	cuteSV
CPU x node	12 x 1	12 x 1	12 x 1	12 x 1
Wall-clock time	12:22:48	03:02:37	1-21:06:15	01:29:37
CPU time	1-19:11:22	1-08:28:26	1-22:42:03	08:10:08
Peak Memory (GB)	63.00	12.62	71.84	3.39
